# Advancing Dry Electroencephalography With Scalable, Soft, and Transcranial Magnetic Stimulation‐Compatible Ti_3_C_2_T_x_ MXene Electrodes for Research and Clinical‐Grade Applications

**DOI:** 10.1002/advs.202511486

**Published:** 2026-02-15

**Authors:** Sneha Shankar, Jakob Michiels, Ksenija Tasich, Ashley Koluda, Ryan Rich, Brian Erickson, Eugenia Angelopoulos, Francesca Cimino, Daryl Hurwitz, Raghav Garg, Spencer R. Averbeck, Doris Xu, Mariam Josyula, Nina Petillo, James J. Gugger, Kathryn A. Davis, John Medaglia, Flavia Vitale

**Affiliations:** ^1^ Department of Bioengineering University of Pennsylvania Philadelphia Pennsylvania USA; ^2^ Center For Neuroengineering & Therapeutics University of Pennsylvania Philadelphia Pennsylvania USA; ^3^ Center For Neurotrauma Neurodegeneration, and Restoration, Corporal Michael J. Crescenz Veterans Affairs Philadelphia Pennsylvania USA; ^4^ Applied Cognitive and Brain Sciences Department of Psychological & Brain Sciences Drexel University Philadelphia Pennsylvania USA; ^5^ Department of Neurology University of Pennsylvania Philadelphia Pennsylvania USA; ^6^ Department of Neurology University of Rochester Rochester New York USA; ^7^ Department of Neurology Drexel University Philadelphia Pennsylvania USA; ^8^ Department of Physical Medicine & Rehabilitation University of Pennsylvania Philadelphia Pennsylvania USA

**Keywords:** bioelectronics, dry EEG electrodes, MXenes

## Abstract

Electroencephalography (EEG), essential for diagnosing and researching neurological disorders, utilizes gelled electrodes, which present limitations in safety, comfort, stability, and usability, particularly in long‐term applications. We introduce a novel dry EEG technology using soft, porous, low‐impedance, Ti_3_C_2_T_x_ MXene electrodes. The 10 Hz impedance of these electrodes across scalp locations is 2.1 ± 1.8 kΩ, comparable to gelled Ag/AgCl electrodes and below clinical thresholds. Ti_3_C_2_T_x_ electrodes maintain stable impedance over 4.5 h on agarose phantoms and retain structure after 50 cycles of 80% axial compression. These electrodes are suitable for simultaneous EEG and transcranial magnetic stimulation (TMS), exhibiting no significant displacement, heating, or unsafe charge densities under TMS fields. We benchmarked dry electrodes across recording scenarios and hair types against gelled electrodes. In full‐scalp steady‐state visual evoked potential (SSVEP) recordings, gelled and Ti_3_C_2_T_x_ electrodes were highly correlated (R > 0.89). Clinical EEG with Ti_3_C_2_T_x_ electrodes captured all features observed with gelled electrodes (R > 0.84) and was rated for clinical quality by neurologists. Furthermore, dry MXene EEG electrode recorded high‐quality EEG for over 4 h. In mobile EEG, Ti_3_C_2_T_x_ electrodes did not induce signal distortions and enabled task‐specific feature detection with a comparable signal‐to‐noise ratio to gelled electrodes. These findings establish dry Ti_3_C_2_T_x_ electrodes as an alternative to gel‐based systems, with broad potential in clinical diagnostics and research.

## Graphical abstract

Dry soft Ti_3_C_2_T_x_ MXene electroencephalography (EEG) electrodes provide low impedance (2.1 ± 1.8 kΩ at 10 Hz), long‐term stability, and enable safe simultaneous EEG and transcranial magnetic stimulation (TMS). Across scalp sites, hair types, and recording paradigms, including steady state visual evoked potentials (SSVEP), clinical EEG, and mobile EEG, they match gelled Ag/AgCl performance (R > 0.8), offering a practical alternative for clinical and research use.

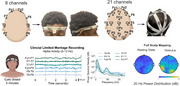

## 1 Introduction

Neurodiagnostic testing is an essential modality in the diagnosis and research of brain disorders, which pose significant public health challenges [1, 2]. Clinical diagnosis, treatment, and monitoring of neurological disorders involve inpatient and at‐home non‐invasive brain imaging and electrophysiological testing via scalp electroencephalography (EEG). EEG is used extensively as the main diagnostic tool for epilepsy, sleep disorders, stroke, and traumatic brain injury, and also finds widespread adoption in human clinical and neuroscience research [3, 4, 5, 6]. Conventional EEG involves placing conductive electrodes on the scalp [7] and capturing the emerging voltage fluctuations generated by the postsynaptic potentials of large populations of nearby cortical neurons [8]. In addition to the low invasiveness, scalp EEG is the method of choice for neurodiagnostics because it offers full‐head coverage, real‐time monitoring and—compared to MRI and other imaging tools—has higher temporal resolution, and it is relatively simple and low‐cost.

Traditional scalp EEG electrodes are made from metals like silver/silver chloride (Ag/AgCl) and gold (Au). To establish a stable and conductive interface with the scalp, metal electrodes require conductive gels and pastes, which also serve as adhesives. As such, these types of electrodes are often referred to as “wet electrodes.” Through the addition of gels and conductive adhesive layers, wet electrodes can establish a conductive, low‐impedance contact between the electrode and the skin across the hair barrier and record high‐quality EEG, but they also have several limitations [9]. Gels and pastes leave residues in the hair, which can be uncomfortable and irritating. Furthermore, vigorous multistep skin preparation involving mechanical abrasion is required to place the electrodes and keep them in contact with the scalp [10]. The placement of wet electrodes is time‐consuming (20‐30 min for the whole scalp) [11] and requires certified EEG technologists, who are not often available outside large clinical centers and in under‐resourced communities [12]. Furthermore, gelled cup electrodes often do not work well with diverse hair types, affecting EEG interpretation and quality. These limitations can lead to delays and disparities in diagnosis and clinical care, affect patient acceptance, and skew the inclusion of patients in certain demographic groups and/or under‐resourced communities in clinical trials and research studies [13]. Additionally, traditional EEG electrodes, and especially metal electrodes, may face issues when used in combination with non‐invasive stimulation approaches such as transcranial magnetic stimulation (TMS), for real‐time and closed‐loop brain state monitoring during non‐invasive neuromodulation, and other brain–computer interface (BCI) applications. Among other non‐invasive neuromodulation methods, TMS is FDA‐approved in US and other countries for the treatment of various neurological conditions such as treatment‐resistant depression, anxious depression, obsessive‐compulsive disorders, migraines, and smoking cessation by generating focused magnetic fields that modulate the excitability of target brain regions. TMS combined with EEG enables real‐time observations of how TMS influences brain activity, optimizing therapeutic protocols and elucidating TMS‐induced neural dynamics [14, 15, 16]. However, some traditional metal EEG electrodes cannot be used with TMS since eddy currents from magnetic pulses could induce heating at the electrode site, posing safety risks to both participants and operators [17]. Specialized TMS‐compatible sintered Ag/AgCl EEG electrodes have been developed, but require wet‐electrode caps, which are equally as cumbersome to set up and remove as conventional devices. These electrodes are manufactured at a standard thickness but could benefit from customizability, allowing for a thinner electrode that permits the stimulation coil to be positioned closer to the scalp, thereby allowing more energy to reach the brain while decreasing machine output to achieve the desired effects.

To circumvent some of these issues there have been efforts to transition to “dry,” i.e., gel‐free electrodes. Dry electrodes can be particularly advantageous for quick EEG setup, rapid and emergency monitoring, and addressing the complexities and discomfort of gels and pastes. Furthermore, dry EEG electrodes can often be reused and are convenient for unsupervised use (i.e., ambulatory and at‐home), as they do not require expert personnel for placement and removal. However, without pastes and gels, it is often difficult to maintain low impedance between the dry electrodes and the scalp, especially in long‐term applications. As such, dry electrodes must rely on other methods to establish a conductive contact with the skin through the hair barrier. A common strategy to circumvent this issue is to use rigid, heavy, bulky headsets that apply high pressure to keep the electrodes on the scalp [18]. However, due to their mechanical rigidity and high contact pressure, these dry EEG headsets are typically quite uncomfortable and are infeasible to use for continuous EEG and sleep monitoring applications. Additionally, headcaps and connectors for these electrodes are often difficult to interface with existing data acquisition systems because they are only compatible with proprietary software and equipment [19]. For some of these systems, the software and equipment use real‐time artifact corrections in order to get usable data, resulting in the raw data, which may be important for analysis, being unavailable to users. These constraints increase the barrier for adoption of novel technologies and limit the versatile use of devices in pre‐existing, well established pipelines in both clinical and research settings. The geometry and materials of dry EEG electrodes also play an important role in the ultimate recording performance and signal quality. Larger electrodes (>1 cm in diameter) and multi‐pin geometries are common design choices for dry EEG, since larger electrode‐skin surface areas result in lower impedance [20].

Dry electrodes are typically made out of metals like Ag/AgCl, Au, or platinum (Pt) due to the durability and excellent DC conductivity of these materials [21, 22, 23, 24]. Metallic dry electrodes, however, require textured pronged geometries to ensure scalp contact and can be quite rigid and uncomfortable, as they exert a large amount of local contact pressure and are not flexible due to the stiff nature of these metals. Furthermore, noble metals, such as Au and Pt, can be expensive to source, process, and manufacture and have high impedance [25]. Conductive polymers such as PEDOT:PSS [26] and hydrogels, which can be characterized as semi‐dry electrodes, have been explored as alternatives to metals for dry electrodes, as they are more flexible while still providing acceptable conductivity [27, 28, 29, 30, 31]. Unfortunately, most of these conductive polymers are moisture‐sensitive, and their performance tends to degrade with time and repeated use [32]. Conductive hydrogels may not be breathable enough for long‐term wear and may require careful cleaning and reapplication or adjustment of the gel [33, 34, 35]. Furthermore, the fabrication process for conductive polymer and hydrogel electrodes can be complex and time‐consuming, as it requires precise control over polymerization or gel formation, electrode fabrication, and connectorization [36]. Finally, polymeric and hydrogel EEG electrodes can be a viable option for use on the skin or minimally hairy parts of the scalp, but their use in conventional full‐scalp EEG montages and diverse hair types is challenging and has not been demonstrated.

More recently, nanoscale materials have been introduced in bioelectronics applications to address the limitations of current electrode technologies. Specifically, 2D materials such as graphene and MXenes have emerged as promising alternatives to metals, polymers, and hydrogels, due to their combination of high conductivity, high capacitance, high surface area, low density, biocompatibility, and ease of functionalization [37, 38, 39]. Graphene has high in‐plane flexibility and mechanical strength, but its integration into large‐area and wearable electrodes is challenging due to the complex synthesis, transfer, processing, and manufacturability [40]. Recently, Ti_3_C_2_T_x_ MXene has been used and validated in a wide range of implantable and large‐scale wearable bioelectronics [41, 42, 43, 44, 45, 46, 47, 48, 49] as well as in regenerative bioelectronic platforms [38]. Compared to graphene, Ti_3_C_2_T_x_ MXene is hydrophilic, stable in aqueous dispersions, and can be processed in liquid phase processing for scalable and high‐throughput device fabrication.

In our previous work, we showed novel approaches to fabricate multiscale dry Ti_3_C_2_T_x_ electrodes for applications in non‐invasive brain, muscle, cardiac, and retinal monitoring, and demonstrated that impedance and recording performance are comparable—and in some cases superior—to state‐of‐the‐art Pt, Au, and Ag/AgCl electrodes [41, 43]. Furthermore, we have demonstrated that Ti_3_C_2_T_x_ electrodes are safe and compatible for use during clinical magnetic resonance imaging (MRI) and computed tomography (CT) [41] and that they can be successfully sterilized with common chemical and thermal processes (i.e., EtO and autoclave, respectively) [50]. We also demonstrated the feasibility of EEG recording during resting state and cognitive tasks from different scalp regions with custom‐designed high‐density (HD) grids of 3 mm dry Ti_3_C_2_T_x_ electrodes (8 or 16 channels) [51]. However, while these HD planar MXene array designs were successful for specific applications and on scalp areas with low curvature and hair density (i.e., forehead, midline, etc.), they required unique custom adapters and surface mount connectors that were not compatible with a variety of standard acquisition systems, and they could not be scaled‐up for full‐scalp and conventional EEG montages, thus limiting their adoption to specific research use cases.

Although MXene electrodes have been previously demonstrated for various electrophysiological recordings, existing approaches have largely focused on planar or textile formats and have not produced a scalable, mechanically compliant dry electrode system that can support full‐scalp EEG, integrate seamlessly with clinical hardware, and be suitable for long‐term use. Prior studies also have not characterized MXene electrodes under multimodal conditions, such as TMS‐EEG or evaluated safety‐critical parameters such as heating, displacement, and secondary current induction. Building on these gaps, in this study, we present the design and validation of novel dry EEG devices utilizing porous Ti_3_C_2_T_x_ MXene electrodes specifically engineered for gel‐free EEG recordings. Our approach employs a scalable and high‐throughput fabrication protocol, enabling the production of soft, flexible, and robust electrodes that maintain low impedance while ensuring consistent and reliable scalp contact. Additionally, we designed and fabricated two custom EEG headsets: one for limited montage applications and another for full‐scalp recordings, demonstrating the versatility of our technology. Moreover, we investigated the safety of Ti_3_C_2_T_x_ MXene electrodes in the context of TMS‐EEG, and assessed electrode heating, displacement, and secondary current induction under repetitive TMS protocols (rTMS) at varying frequencies and field strengths. Finally, we demonstrate the ability to record high‐quality, artifact‐free EEG with dry Ti_3_C_2_T_x_ MXene headsets on diverse hair types and in several realistic use‐case scenarios, including human cognitive research settings, clinical EEG monitoring, and wireless mobile EEG, benchmarking against commercial Ag/AgCl electrodes. Our work represents a significant advancement in EEG technology, combining novel materials science with rigorous safety and performance testing to deliver a scalable, high‐performance, inclusive, and translatable solution for dry EEG recordings.

## 2 Results and Discussion

### 2.1 Rapid, Scalable Fabrication of Dry, 3D Ti_3_C_2_T_x_ MXene Electrodes

**FIGURE 1:**
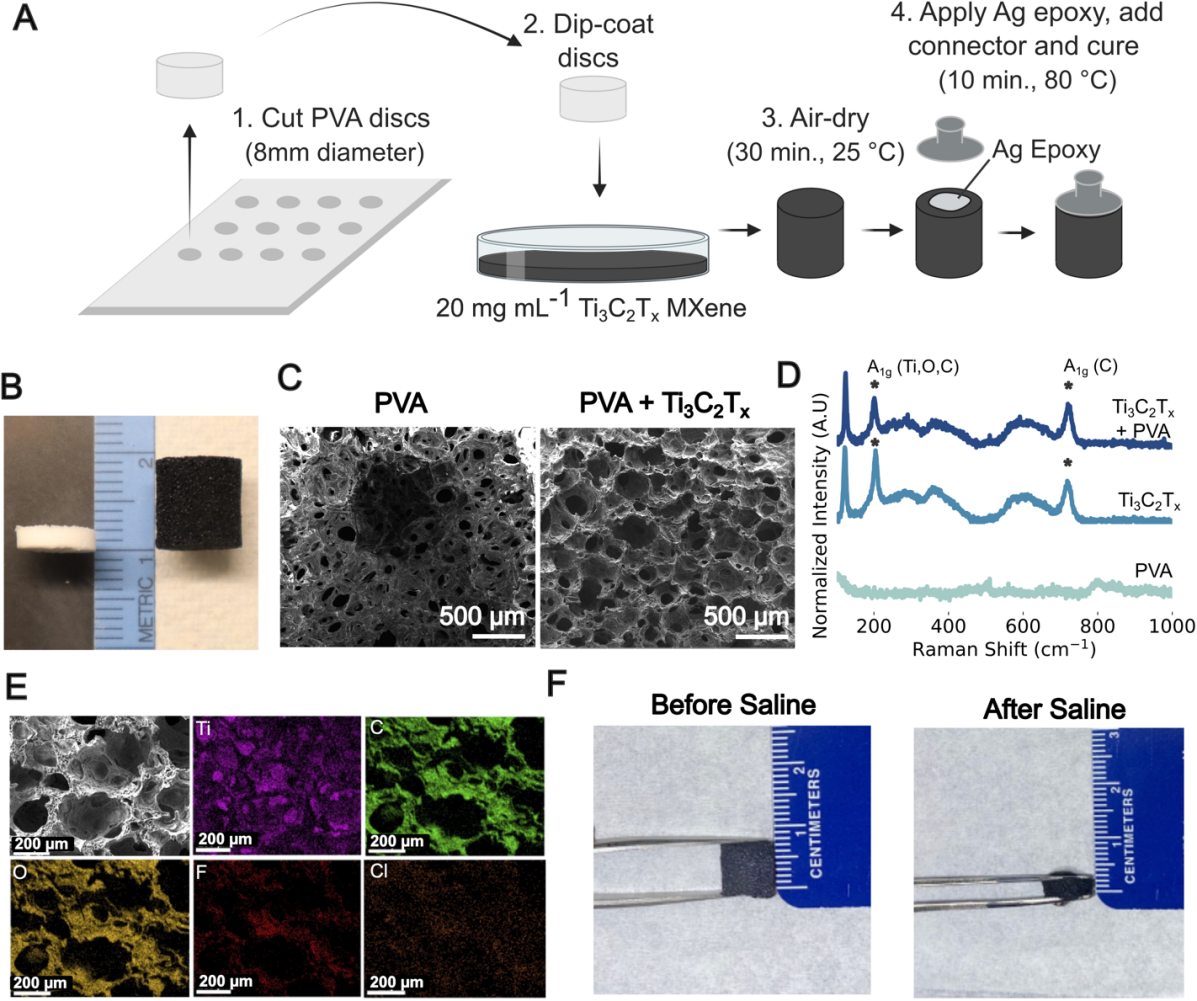
(A) Fabrication of dry Ti_3_C_2_T_x_ MXene EEG electrodes (Created in BioRender. Lab, V. (2026)). (B) Axial expansion of the PVA templates dip‐coating in the Ti_3_C_2_T_x_ dispersion. (C) SEM images of PVA discs before and Ti_3_C_2_T_x_ coating (100× magnification). (D) Raman spectra of PVA discs, Ti_3_C_2_T_x_ films drop cast on glass, and Ti_3_C_2_T_x_‐PVA electrodes. (E) EDX results for dry Ti_3_C_2_T_x_ MXene EEG electrodes. (F) Compression of Ti_3_C_2_T_x_ electrodes (left) dry, and (right) after absorption of 1 mL of saline.

To form the templates of soft, 3D dry electrodes, we start from an absorbent sheet of hydroxylated polyvinyl alcohol (PVA, Medtronic Kennedy Sinus‐Pak) and cut out 8 mm disks (Figure 1A). We chose PVA because it is biocompatible, hydrophilic, and it is already commonly used in different medical devices, including contact lenses, arterial grafts, wound dressing, and osteochondral grafts [52]. We then dip‐coat the PVA disks in an aqueous dispersion of Ti_3_C_2_T_x_ MXene at a concentration of 20 mg mL^−1^. Owing to its hydrophilic nature, upon water absorption, the PVA aerogels expand axially from their initial disk structure (height: ∼ 1 mm) to a cylindrical shape (height: ∼8 mm), while the diameter remains unchanged (Figure 1B). Scanning electron microscopy (SEM) imaging of the PVA disks (Figure 1C) shows the expansion and increase in porosity of the PVA matrix (initial pore size: 72 ± 23 µm, final pore size: 213 ± 25 µm) upon absorption of the Ti_3_C_2_T_x_ dispersion, with Ti_3_C_2_T_x_ flakes homogeneously coating the PVA template (Figure S1). Energy‐dispersive X‐ray spectroscopy (EDX) analysis of the Ti_3_C_2_T_x_‐PVA electrodes shows predominant ‐Ti and ‐C as well ‐O peaks (Atomic%: Ti: 24.76, C: 39.45, O: 35.14), alongside with negligible amount of ‐F and ‐Cl surface groups (Atomic %: F: 0.19, Cl: 0.47) from the etching phase of the Ti_3_C_2_T_x_ synthesis. The %‐O content of the Ti_3_C_2_T_x_‐PVA electrodes (Figure S2) is comparable to that of pristine PVA (Atomic % O – 40.04), which suggests the lack of oxidative damage to Ti_3_C_2_T_x_ from the fabrication process. Raman spectra of the Ti_3_C_2_T_x_‐PVA electrodes excited with a 785 nm laser show the out‐of‐plane A_1g_ vibration modes of Ti, C, and O atoms at ∼200 cm^−1^ and of the C atoms at ∼ 720 cm^−1^ (Figure 1D). These characteristic Raman modes of Ti_3_C_2_T_x_ [53] can also be observed on dry Ti_3_C_2_T_x_ films on glass substrates, but not on the PVA templates, confirming that the templates are uniformly covered by Ti_3_C_2_T_x_ [53] flakes and the preservation of Ti_3_C_2_T_x_ quality. The final Ti_3_C_2_T_x_ electrodes initially present as firm structures but then soften in the presence of moisture (after the absorption of approximately 1 mL of saline) (Figure 1E and F). After drying the Ti_3_C_2_T_x_‐PVA electrodes at room temperature for 45 min, Ag/AgCl button snap connectors are attached with Ag epoxy, followed by a final 10 min curing step in the oven at 80°C. The total manufacturing time for a batch of 10 electrodes is <1 h, including drying. It is worth noting that this process can be implemented on any porous and hydrophilic aerogel material and scaled up with die or laser cutting of the PVA templates, further enhancing manufacturability and scalability.

### 2.2 Fabrication of Dry MXene EEG Headsets

**FIGURE 2:**
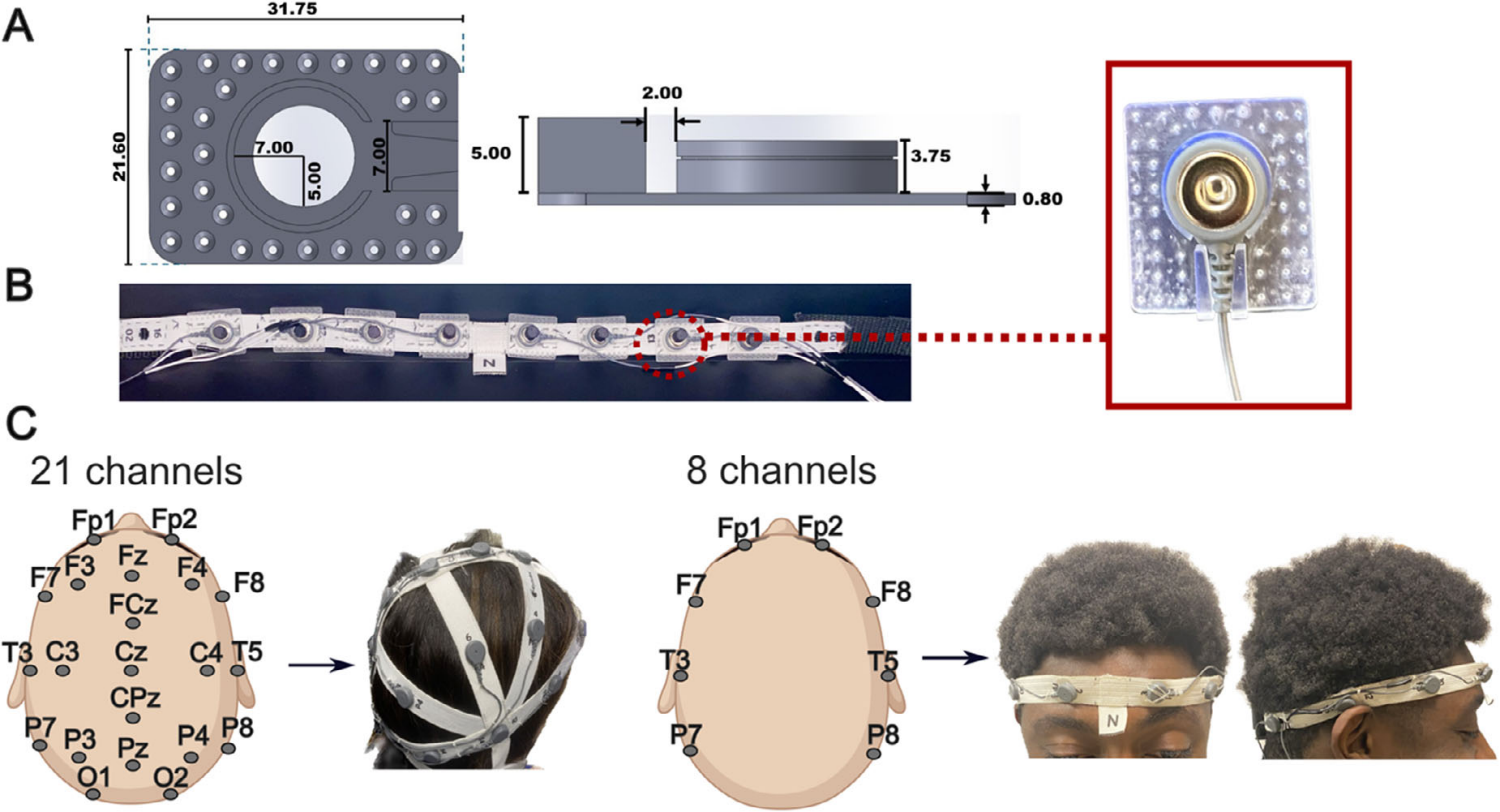
(A) Schematic of the custom 3D printed connector holders with dimensions. (B) Photograph of the holders with attached electrodes and connectors. (C) Left: 10–20 EEG headsets with 21 electrodes on defined anatomical landmarks marked on the channel mapping schematics; right: 8‐electrode headband with channel mapping (Created in BioRender. Lab, V. (2026)).

In standard clinical brain monitoring and diagnostics, a set of 21 EEG electrodes is placed at defined cranial landmarks and at specific relative distances [54]. This internationally accepted configuration, known as the 10–20 system, ensures consistent and repeatable placement of the electrodes across individuals, scalp sizes, and clinical centers. To replicate this standard configuration, we built custom headsets using flexible elastic bands with 21 electrode connectors placed in correspondence with the 10–20 EEG system scalp locations. The connectors consist of snap‐on leads that are held in place by custom 3D‐printed holders, which allow for keeping the leads securely in place even during movement and easily removing them as needed (Figure 2A–C). Using button snaps for the EEG connectors offers several key advantages. First, these connectors are standard in EEG systems and are compatible with touch‐proof leads used in several commercial EEG amplifiers (both for clinical and research use), simplifying the integration of these novel dry electrodes with existing equipment. Second, these connectors are easy to use and allow for quick and secure electrode placement/removal, providing the robustness and efficiency required in clinical settings. Finally, the versatility of these connectors—also available in MRI‐compatible versions—enables using our dry MXene EEG electrodes in different research and clinical scenarios, making them cost‐effective and lowering the barrier for adoption. Leveraging the versatility of the electrode and headset fabrication, we also designed a reduced‐montage EEG headband consisting of 8 electrodes placed at defined anatomical locations along the scalp circumference (Fp1, Fp2, F7, F8, T7, T8, P7, and P8) (Figure 2B, C). Similar to the full‐head design, the electrodes are secured to the headband with 3D‐printed holders and plugged into the amplifier via flat snap connector leads (Figure 2B). Both the headband and 10–20 EEG cap are fabricated from an elastic fabric and equipped with an adjustable band to secure the headsets to different head sizes and ensure that the electrodes can safely contact the scalp. The headset designs also allow for easy access to electrode sites for adjustment and replacement.

### 2.3 Electrochemical and Mechanical Characterization of Dry Ti_3_C_2_T_x_ EEG electrodes

**FIGURE 3:**
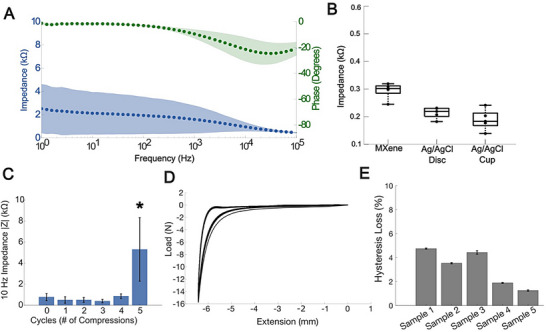
(A) Average impedance modulus and phase spectra, with shaded region representing standard deviation, for 6 electrodes (n = 3 participants). (B) Average 10 Hz impedance of dry Ti_3_C_2_T_x_ and gelled electrodes on agarose phantoms (n = 5 electrodes). (C) 10 Hz impedance throughout 5 cycles of compression/extension, presented as mean ± SD, n = 5 electrodes, P‐values calculated using a two‐tailed Mann–Whitney U test (p = 0.03; *P<0.05). (D) Load and extension curves over 50 compression cycles for n = 1 electrode. (E) Average hysteresis loss of n = 5 different dry Ti_3_C_2_T_x_ electrodes over 50 compression cycles.

The electrode‐skin interface impedance represents the resistance to charge and signal transfer at the interface between an EEG electrode and the skin. Such interfacial impedance is critical for the quality, fidelity, and stability of the EEG signal [55, 56]. Some of the key factors affecting impedance are the electrode material, the quality of contact between the skin and the electrode, skin conditions and preparation, and the application of conductive gels and adhesives [56]. Compared to conventional gelled electrodes, dry electrodes usually exhibit higher impedance (i.e., >50 kΩ at 10 Hz) [57], and may experience inconsistent contact with the scalp as they do not rely on conductive gels or pastes and are typically rigid in nature. Such high impedance and contact instability can compromise signal fidelity, increase noise, and introduce artifacts [58]. To measure the electrode‐skin impedance of dry Ti_3_C_2_T_x_ EEG electrodes, we conducted electrochemical impedance spectroscopy (EIS) analysis on the scalp of 3 participants. For each participant, we prepped the skin with a simplified non‐irritating preparation protocol (Figure S3), then we acquired EIS with dry Ti_3_C_2_T_x_ electrodes in two different locations: 1) on the lateral forehead Fp1, a region with no hair, and 2) on the temporal region T8, a site with hair. For the measurement, we used gelled Ag/AgCl electrodes on the mastoid as a reference. Figure 3A shows the average EIS spectra in the 1–10^5^ Hz range for 6 electrodes (n = 2 for each participant). We found that at 1–100 Hz—the relevant EEG frequency band—the impedance modulus of the dry Ti_3_C_2_T_x_ EEG electrodes shows a frequency‐independent response with a phase shift <20°. This indicates that the dry Ti_3_C_2_T_x_ electrodes’ response is predominantly resistive and that they can collect and transduce the EEG signal without introducing phase distortions [59]. Maintaining signals without phase distortions is particularly imperative in applications such as cognitive studies and TMS‐EEG that rely on phase information for analysis and intervention [60, 61]. At 10 Hz, the average impedance across our cohort of participants with a diversity of hair types was 2.1 ± 1.8 kΩ. This value is in agreement and well within the range of what is considered acceptable for clinical‐grade gelled EEG electrodes for human use (i.e., <10 kΩ) [62]. To benchmark the performance and impedance stability of dry Ti_3_C_2_T_x_ electrodes over time, we collected EIS continuously for 4.5 h on an agarose phantom and compared it with two different types of Ag/AgCl gelled electrodes of common use: 20 mm discs and 3 mm cups (n = 5 electrodes of each type) (Figure S4). Disk electrodes have a larger, flat surface and are often used in research settings, whereas cup electrodes are smaller, concave, and are designed to be filled with conductive gels for long‐term monitoring (e.g., inpatient continuous EEG in the epilepsy monitoring unit) [20, 62]. At the beginning of the study, the 10 Hz impedance of dry Ti_3_C_2_T_x_ electrodes was 0.29 ± 0.03 kΩ, which was slightly higher than the starting impedance of both gelled disc (0.21 ± 0.02 kΩ) and cup electrodes (0.18 ± 0.04 kΩ) (Figure 3B). The impedance of dry Ti_3_C_2_T_x_ electrodes increased by ∼0.2 kΩ for the first 2 h, then stabilized and remained unchanged for the following 2.5 h (Z_2hrs._ 0.42 ± 0.04 kΩ, Z_final_ 0.48 ± 0.07 kΩ), while the impedance of both types of gelled electrodes remained essentially unchanged for the entire duration of the study (Z_2hrs._ disc: 0.19 ± 0.02 kΩ, Z_final_ disc: 0.20 ± 0.03 kΩ, Z_2hrs._ cup: 0.16 ± 0.03 kΩ, Z_final_ cup: 0.16 ± 0.03 kΩ) (Figure S5). The rise in impedance of dry Ti_3_C_2_T_x_ electrodes may be attributed to the lack of gel underneath the dry electrodes, while the Ag/AgCl electrode contact area remained hydrated due to the agarose gel. However, this impedance change was minimal and remained well below the standard clinical impedance thresholds (10 kΩ) for EEG electrodes.

In addition to monitoring the stability of impedance over time, EIS measurements were also collected in a standard three‐electrode electrochemical cell for equivalent circuit modeling. Bode plots from this setup, averaged across ten electrodes show a remarkably low and stable impedance across all frequencies, with only a very slight increase in the average impedance at 1 kHz (206 ± 25 Ω) compared to 10 Hz (267 ± 36 Ω, Figure S6A). The stable impedance magnitude is clearly represented in the near‐zero degree phase magnitude across all frequencies (Figure S6B).

Further, by modeling the Ti_3_C_2_T*_x_* electrodes as a Randles cell with parallel RC components for both the interface as well as the porous membrane of the Ti_3_C_2_T*_x_* MXene electrodes and fitting the model to the measured Nyquist plots, we determined the key equivalent circuit parameters such as the solution resistance (R_s_), the charge‐transfer resistance (R_ct_), and the double‐layer capacitance (C_dl_) (Figure S6C). From fitting the EIS data with the equivalent circuit model of a porous electrode, we found that the solution resistance was within the expected theoretical range, falling between the values for pure saline and dry conductive agarose (72 Ω < R_s_ < 250 Ω, Supplemental Table S1) [63]. Additionally, the charge‐transfer resistance of the electrodes was remarkably low, and the double‐layer capacitance was quite high, even when normalized by surface area (R_ct_: 340.4 ± 69.5 Ω; C_dl_: 552.4 ± 383.7 µF cm^−2^). These values align well with our understanding of Ti_3_C_2_T*_x_*—especially in such a porous form factor—as a highly conductive and capacitive material, capable of facilitating exceptionally rapid kinetic charge transfer and capacitive charging and discharging at the electrode/skin interface [64, 65]. Conductivity was measured on rectangular MXene/PVA samples (n = 4, 1.15 × 0.70 × 0.50 cm), yielding an average conductivity of 1.079 ± 0.852 S m^−1^ (measured perpendicular to the width) and 0.947 ± 0.440 S m^−1^ (measured perpendicular to the length), suggesting even deposition of MXene and uniform conductivity throughout the porous substrate.

In addition to favorable electrochemical characteristics, EEG electrodes need to be mechanically robust enough to endure the forces that are applied during placement and removal. This is particularly relevant to dry Ti_3_C_2_T_x_ electrodes since their foam‐like porous structure is highly compressible. Here, we compressed n = 5 Ti_3_C_2_T_x_ electrodes by 80% of their axial length (∼6 mm) with a 15 N load cell and collected EIS on an agarose phantom after each cycle of compression/extension. After 5 cycles, the 10 Hz impedance of Ti_3_C_2_T_x_ showed a statistically significant increase from its initial value (Z_initial_ = 0.3 ± 0.03 kΩ, Z_final_ = 5.3 ± 3.0 kΩ; Mann‐Whitney U test, p = 0.03, Figure 3C) but remained within the acceptable limit of 10 kΩ. This increase may have occurred in response to nanoscale delamination of MXene flakes within the PVA matrix through the compression cycles. After 50 compression cycles, the Ti_3_C_2_T_x_ electrodes retained the ability to extend back to the original height and did not show any visible mechanical degradation in their load‐extension profiles (Figure 3D), which show a hysteresis loss <5% across 50 deformation cycles (Figure 3E). Such a low hysteresis loss here indicates that our Ti_3_C_2_T_x_ MXene electrodes have a stable energy dissipation, behave elastically, and maintain their integrity under 50 cycles of repeated loading and unloading.

### 2.4 TMS Safety of Dry Ti_3_C_2_T_x_ EEG Electrodes

**FIGURE 4:**
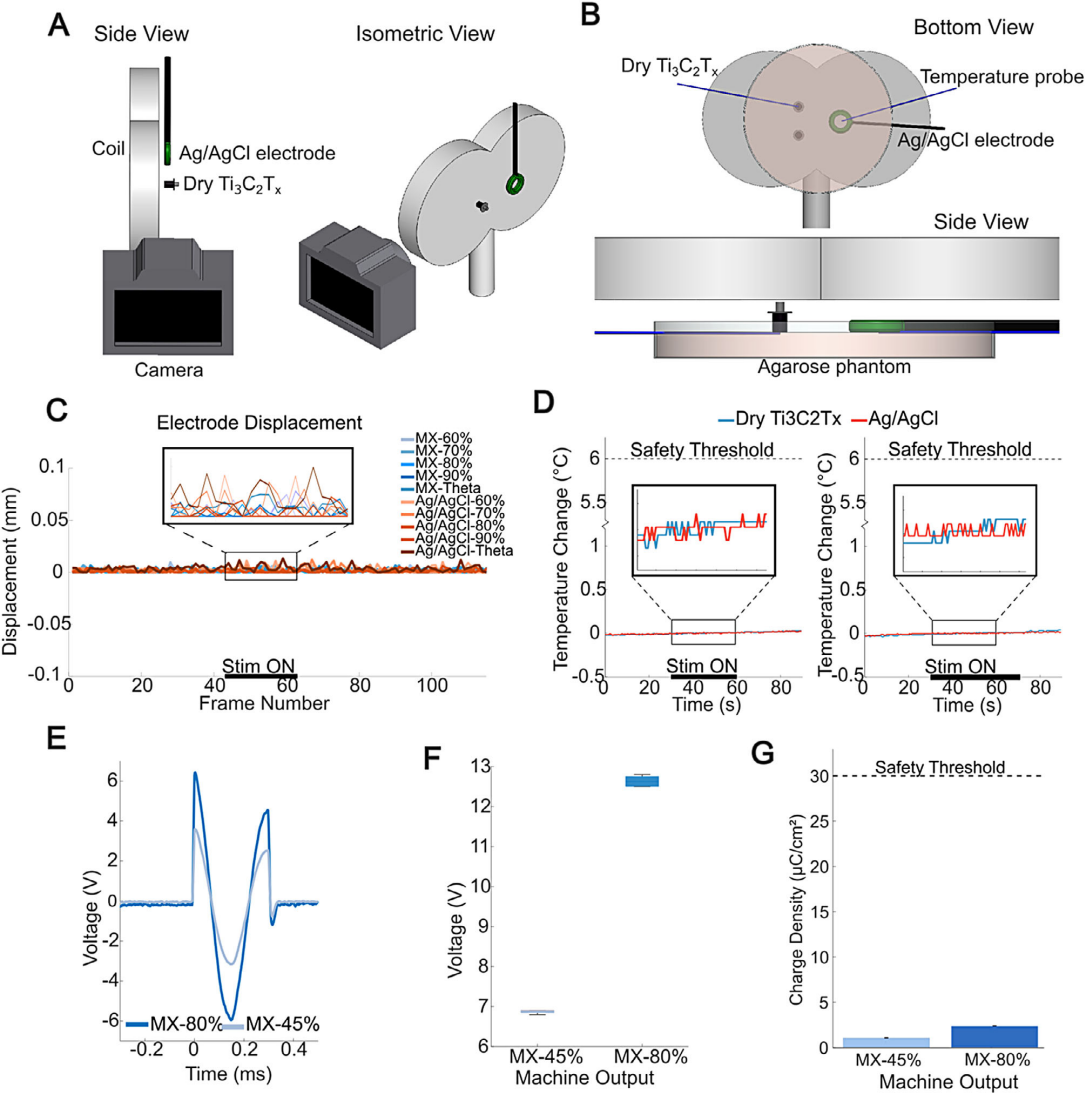
(A) TMS‐induced displacement and (B) temperature and voltage measurement setups (Created in BioRender. Lab, V. (2026)). (C) Displacement from a single pulse stimulation at varying TMS machine output (n = 1 per stimulation condition). (D) Temperature profiles measured before, during, and after (left) 10 Hz TMS stimulation at 80% output and (right) 50 Hz theta burst at 45% output (n = 2 for stimulation condition). (E) Biphasic voltage pulses on the dry Ti_3_C_2_T_x_ electrodes with (F) peak‐to‐peak amplitudes and (G) resulting charge densities (n = 3 for stimulation condition). All percentages denote the TMS machine output.

Safety concerns arise when traditional EEG electrodes are used in conjunction with TMS due to the interaction between TMS‐generated magnetic fields and the conductive materials in these electrodes. Specifically, adverse effects such as electrode displacement, heating, and secondary currents can occur and compromise patient safety and EEG recording quality [17, 66]. Therefore, it is critical to establish the safety profiles of electrodes under TMS conditions and ensure their compatibility and reliability for use in TMS‐EEG applications. Here, we investigated the TMS safety of dry Ti_3_C_2_T_x_ electrodes and benchmarked against commercial TMS‐compatible split‐ring type electrodes commonly used in TMS‐EEG (Ag/AgCl B18 model from EasyCap), (Figure 4A,B). In this study, we adopted two experimental setups. For displacement testing, a dry Ti_3_C_2_T_x_ electrode and an Ag/AgCl electrode were suspended in air side‐by‐side, 1 mm from the TMS coil (Figure 4A). A Canon EOS M50 high‐definition digital camera was positioned parallel to the electrodes to capture and then quantify displacement. The electrodes were subjected to two different TMS protocols: single pulses delivered at 60%, 70%, 80%, and 90% machine output (MO), and 50 Hz theta burst stimulation at 45% MO for 10 s [67]. No measurable movement was detected in both dry Ti_3_C_2_T_x_ electrodes and Ag/AgCl electrodes under any of the investigated TMS protocols (Figure 4C).

For temperature and induced voltage and charge testing, two adjacent Ti_3_C_2_T_x_ electrodes and one Ag/AgCl electrode were mounted on a conductive 0.6% agarose phantom, and the temperature at the electrode surface was monitored with a fiber optic probe placed between the electrodes and the agarose phantom. Electrode voltage was continuously measured during 10 Hz TMS stimulation at 80% MO and a 50 Hz theta burst sequence at 45% MO, both delivered over 30 s intervals. The maximum temperature increase during both TMS protocols on the dry Ti_3_C_2_T_x_ MXene electrode was 0.08 ± 0.02 °C (initial temperature: 18.26 ± 0.67 °C, final temperature: 18.34 ± 0.65 °C), well below the 6 °C safety threshold recommended for devices in contact with the skin during neurostimulation (Figure 4D). [68, 69, 70] The Ag/AgCl electrode also showed no temperature changes (initial temperature: 17.85 ± 0.69 °C, final temperature: 17.89 ± 0.69°C) (Figure 4D). As for the induced voltage from TMS, the 10 Hz TMS at 80% output and the 50 Hz theta burst TMS at 45% output produced 12.63 ± 0.14 and 6.76 ± 0.06 V, across adjacent Ti_3_C_2_T_x_ electrodes, respectively (peak‐to‐peak values; Figure 4E, F). The corresponding charge densities on the electrodes, calculated with Equation (3), were 2.87 ± 0.05 µC cm^−2^ (10 Hz at 80% output) and 1.35 ± 0.01 µC cm^−2^ (50 Hz theta burst at 45% output; Figure 4G), which are in both cases well below the 30 µC cm^−2^ safety limit to prevent tissue damage [69]. Excessive charge densities can cause electrolytic tissue damage and lead to burns [60]. Minimal heating of MXene electrodes also presents a potential advantage for blinding in neuromodulation studies. In some studies, sham conditions are designed to minimize or eliminate heating, which can otherwise serve as a sensory cue that compromises subject blinding. By demonstrating that MXene electrodes produce very low heating, participants are less likely to detect differences in thermal sensation between active and sham stimulation, improving the robustness of blinded study designs [71].

### 2.5 Full Scalp EEG With Dry Ti_3_C_2_T_x_ MXene Electrodes

**FIGURE 5:**
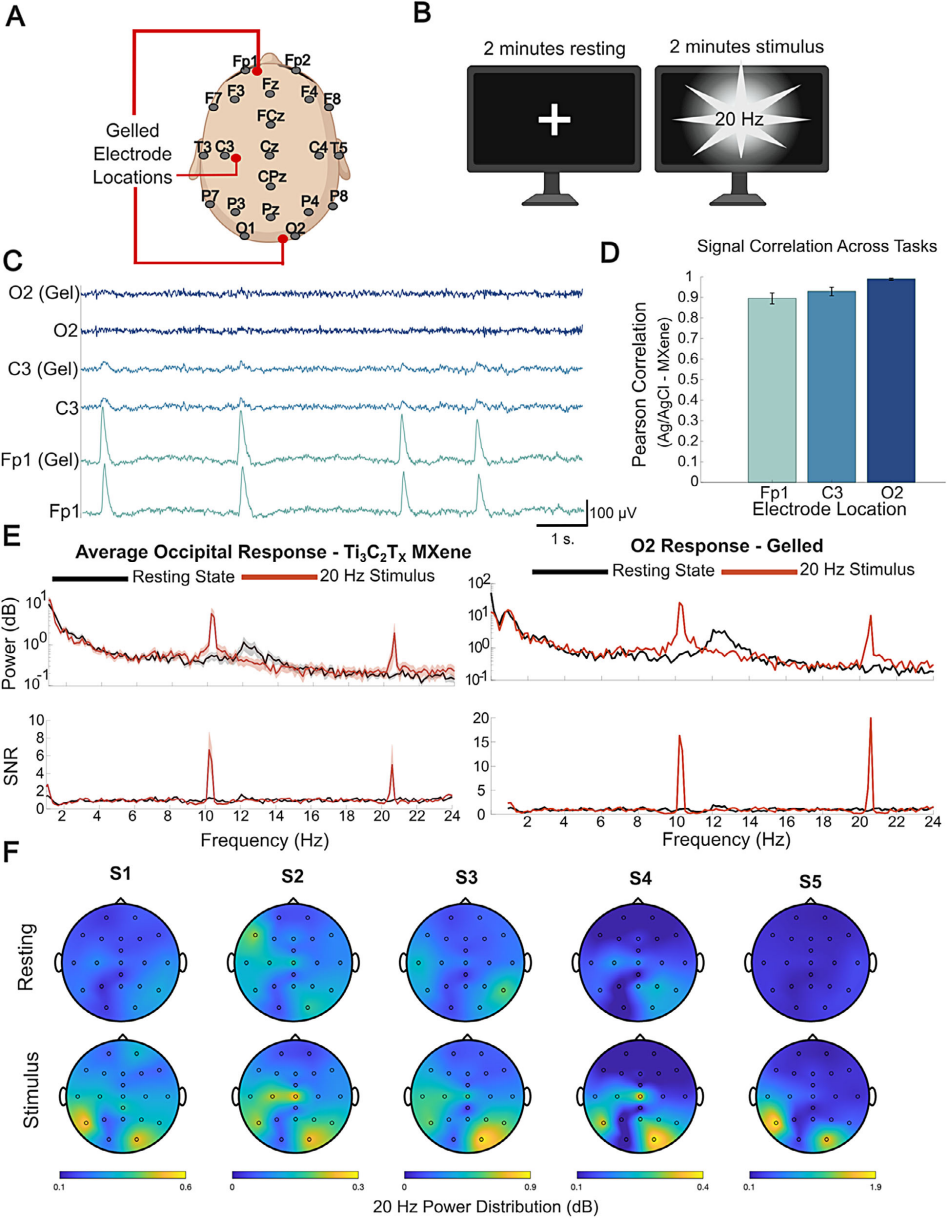
(A) Schematics of the dry Ti_3_C_2_T_x_ and Ag/AgCl cup electrode positions. (Reference and ground electrodes on the mastoids, not shown). (B) Schematics of the visual stimulation presentation setup (Created in BioRender. Lab, V. (2026)). (C) Representative 10 s of EEG from n = 1 participant on dry Ti_3_C_2_T_x_ and adjacent Ag/AgCl electrodes. (D) Pearson correlation presented as mean ± SD for electrode pairs across brain regions calculated using a paired t‐test (α = 0.05; p = 0.01) across all n = 5 participants. (E) Representative average (top) PSD and (bottom) 20 Hz SNR during resting and stimulus recordings across (left) all occipital/parietal MXene electrodes (P3, P4, O1, O2, Pz) and (right) the most proximal gelled electrode at O2 in subject 5 (S5). (F) Topographical maps of EEG power distribution at 20 Hz at resting state vs. stimuli for all n = 5 participants (Subject 1 – S1, Subject 2 – S2, etc.).

Consistent and comfortable placement of the electrodes on the scalp is key to ensuring repeatability, reliability, and quality of EEG recordings. Ag/AgCl gelled electrodes are widely used for full scalp EEG and are useful in ensuring proper adhesion between the electrodes and the scalp due to the presence of gel, while minimizing subject‐to‐subject scalp variability and impedance. Due to the lack of such gels, consistent and comfortable placement of dry EEG in addition to acquiring adequate signal is significantly more challenging [72], especially when attempting to record from the entire scalp. Here, we sought to validate and benchmark the 21‐channel dry Ti_3_C_2_T_x_ EEG headset (i.e., 10–20 system) against commercial Ag/AgCl gelled electrodes during common tasks performed in cognitive research and BCI paradigms to ensure we could record EEG across the full scalp. In these studies, we asked participants (n = 5) to wear the 10–20 dry Ti_3_C_2_T_x_ headcap placed using a minimal skin preparation protocol (Figure S3). Then, we placed 3 additional Ag/AgCl cup electrodes (3 mm diameter, Figure S4) adjacent to 3 dry electrodes for direct comparison of signals across scalp sites with varying levels of hair density: Frontal (Fp1), Motor (C3), and Occipital (O2) (Figure 5A). Reference and ground Ag/AgCl electrodes were placed on the mastoid. Then, we checked the impedance to ensure that all electrodes were <100 kΩ and showed a stabilized baseline signal on the Bittium NeurOne amplifier. We collected 2 min of resting state EEG, then asked participants to observe a screen with a 20 Hz flashing stimulus source for 2 min (Figure 5B). These flashing visual stimuli are expected to elicit a 20 Hz steady‐state visual evoked potential (SSVEP) response [73], which has been widely accepted for BCIs and noted for its accuracy [74].

EEG collected on dry Ti_3_C_2_T_x_ electrodes was highly comparable to adjacent Ag/AgCl sites, as also indicated by Pearson correlations > 0.8 throughout the whole recording session (i.e., resting state + SSVEP task, Fp1: 0.895 ± 0.059, C3: 0.929 ± 0.047, O2: 0.989 ± 0.011; n = 5 electrode pairs; Figure 5C,D). In the SSVEP task, across all participants, we observed the expected increase in the power spectral density (PSD) and signal‐to‐noise ratio (SNR) of the EEG collected on dry Ti_3_C_2_T_x_ electrodes centered at the frequency of the visual stimulation (i.e., 20 Hz, Figure 5E and Figure S6). Specifically, we found that the increase in SNR at 20 Hz compared to resting states of electrodes in the region of interest (P3, P4, Pz, O1, O2) was significant across all participants (two‐tailed paired t‐test, p = 0.01 ± 0.01). This increase in the 20 Hz response is also observed on the most proximal gelled Ag/AgCl electrode placed in the occipital region. Topographical maps of the 20 Hz power distribution on the scalp built from the full‐scalp dry EEG recordings (Figure 5F) show that the 20 Hz activity was spatially localized on the occipital electrodes, which are the most proximal to the visual cortex. Overall, these results demonstrate the ability to record full‐scalp EEG with a 10–20 dry Ti_3_C_2_T_x_ headset designed to optimize scalp contact and user comfort. Furthermore, the full‐scalp dry Ti_3_C_2_T_x_ headsets can collect EEG signals of the same quality and reliability as state‐of‐the‐art commercial Ag/AgCl electrodes and capture relevant EEG features for cognitive and BCI research applications.

### 2.6 Clinical and Long Term EEG Recordings With 8‐Channel Dry Ti_3_C_2_T_x_ EEG Headband

**FIGURE 6:**
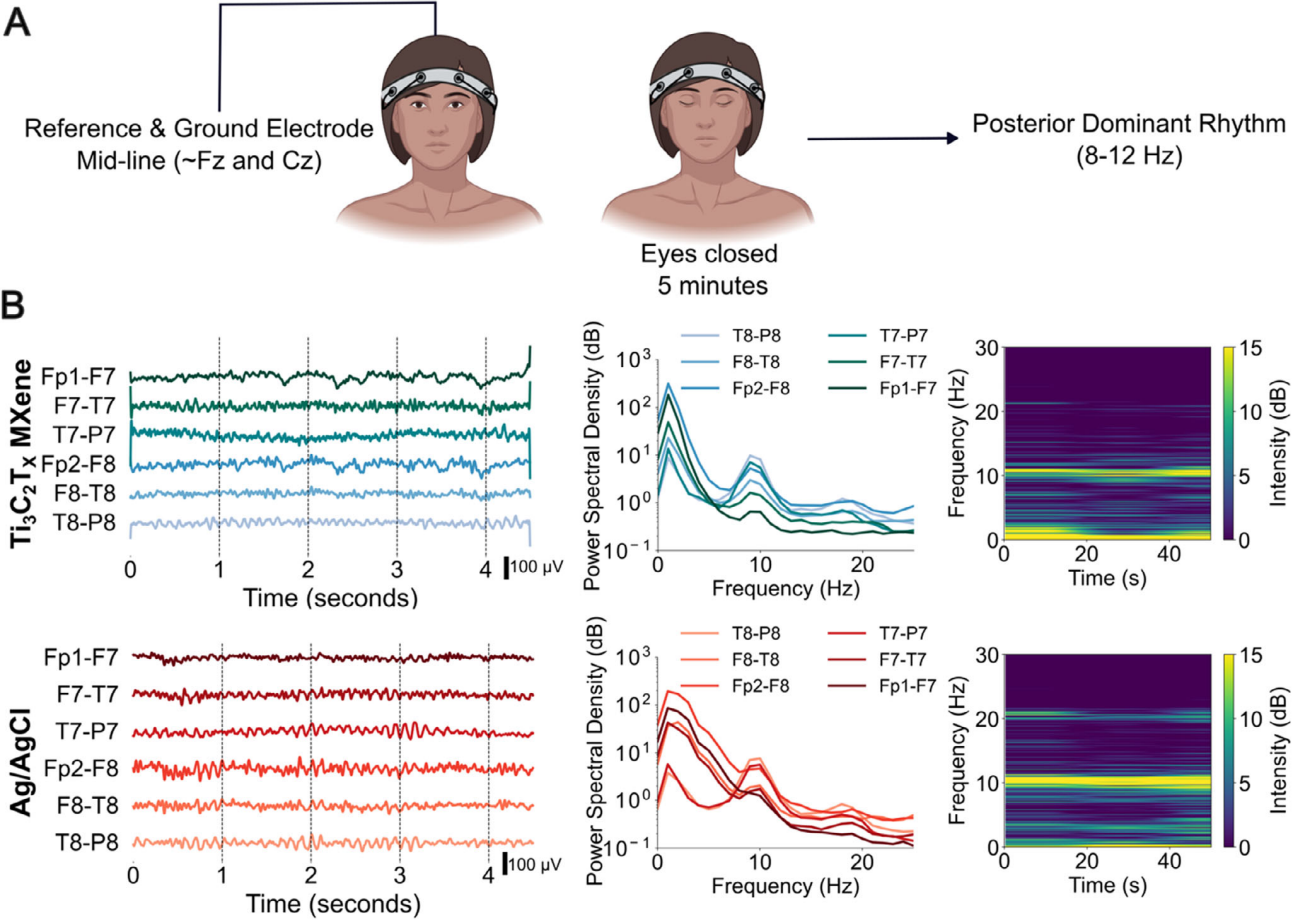
(A) Schematics of the setup and task for clinical EEG recordings with the 8‐channel dry Ti_3_C_2_T_x_ electrode headband (Created in BioRender. Lab, V. (2026)). (B) From left: Representative 4 s timeseries (bipolar montage), PSD, and spectrogram of the EEG recorded with (top) dry Ti_3_C_2_T_x_ and (bottom) Ag/AgCl cup electrodes for participant S3 (PSDs of all other participants are in Figure S8).

Reduced montage configurations are becoming increasingly popular for rapid bedside and at‐home EEG monitoring. Specific to clinical settings, such as in the intensive care unit (ICU), reduced montage EEG has proven useful for quick diagnostics and detection of slowing brain patterns in patients with brain injury who present with symptomatic seizures [75, 76, 77]. With these scenarios in mind, we sought to validate the 8‐channel dry Ti_3_C_2_T_x_ EEG headbands in a relevant clinical application. We recruited and consented n = 5 patients who were scheduled for a routine EEG exam in the outpatient EEG clinic of the Hospital of the University of Pennsylvania and conducted the study with the dry Ti_3_C_2_T_x_ headband prior to their EEG visit. The dry EEG headband was placed on patients on supine position by research personnel using a limited skin preparation protocol (Figure S3), with reference and ground‐gelled Ag/AgCl electrodes placed on the scalp midline by an EEG technician. The leads for the electrodes, both dry and gelled, were plugged into the clinical EEG amplifier used for diagnostics and monitoring (Natus). The total setup time took about 5 min. After placing the dry EEG headband, we asked patients to rest with open and closed eyes for 2 to 3 min at a time for each condition and collected the EEG (Figure 6A). We designed the eyes open/closed tasks to match the clinical protocol, which allowed us to compare the EEG collected in the research (i.e., dry Ti_3_C_2_T_x_) and clinical (i.e., Ag/AgCl cup) sessions on the same patient. Each recording session was roughly 20 to 30 min total. A comparison of the signals shows that the EEG on dry Ti_3_C_2_T_x_ electrodes is again highly comparable to gelled Ag/AgCl, the current standard in clinical EEG (Figure 6B). Then, we looked at the posterior dominant rhythm (PDR) assessed as an increase in the power of the EEG at 8–12 Hz (Alpha frequency band) in the eyes‐closed resting state condition. The PDR is typically observed in the occipital regions of the brain and is a key component of the resting EEG that reflects normal cortical activity when the individual is awake and relaxed. Measuring PDR is crucial for several reasons: 1) PDR is a baseline indicator of cortical function, 2) it helps assess the integrity of the sensory and cognitive processing areas, and 3) it provides insights into overall brain health. Deviations or abnormalities in PDR can be indicative of various neurological conditions, including epilepsy, sleep disorders, and cognitive impairments [78]. Here, during the eyes‐closed task, we detected PDR in the EEG acquired with both sets of electrodes (Figure 6B and Figure S8). Looking at the PSD of the EEG, we also found the expected spatial distribution of the PDR response, with posterior electrodes (i.e., T‐P dipoles) having a higher PDR response compared to anterior electrodes (i.e., Fp‐F). To compare the similarity (or lack thereof) of the EEG collected on each of the dry Ti_3_C_2_T_x_ electrodes to that on the Ag/AgCl in the corresponding location, we calculated the Pearson correlation of the PSD. We found that the EEG were highly correlated in all the locations and in all 5 patients (T8‐P8: 0.85 ± 0.07, F8‐T8: 0.84 ± 0.04, Fp2‐F8: 0.79 ± 0.1, T7‐P7: 0.84 ± 0.05, F7‐T7: 0.93 ± 0.02, Fp1‐F7: 0.91 ± 0.04, Figure S8). Furthermore, a board‐certified neurologist read the EEGs collected with both electrode types and confirmed that all key features seen on the clinical EEG (i.e., PDR, symmetry, blink artifacts, frequency, and amplitude) were also present on the dry Ti_3_C_2_T_x_ electrode recordings and that the dry EEGs were of acceptable clinical quality. Ensuring that EEG data obtained from dry Ti_3_C_2_T_x_ electrodes is qualitatively interpretable by a clinician on their clinical EEG visualization software is important and confirms that 1) the signal collected on our novel dry electrodes accurately represents the brain activity, and 2) the dry EEG headband can be seamlessly integrated with current clinical software and workflows.

**FIGURE 7:**
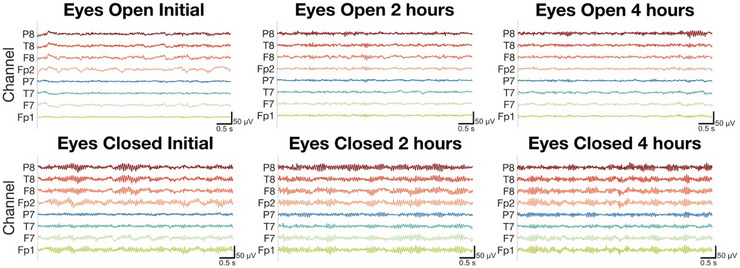
Representative 10 s EEG traces during eyes‐open (top) and eyes‐closed (bottom) tasks for subject DYOX.

**FIGURE 8:**
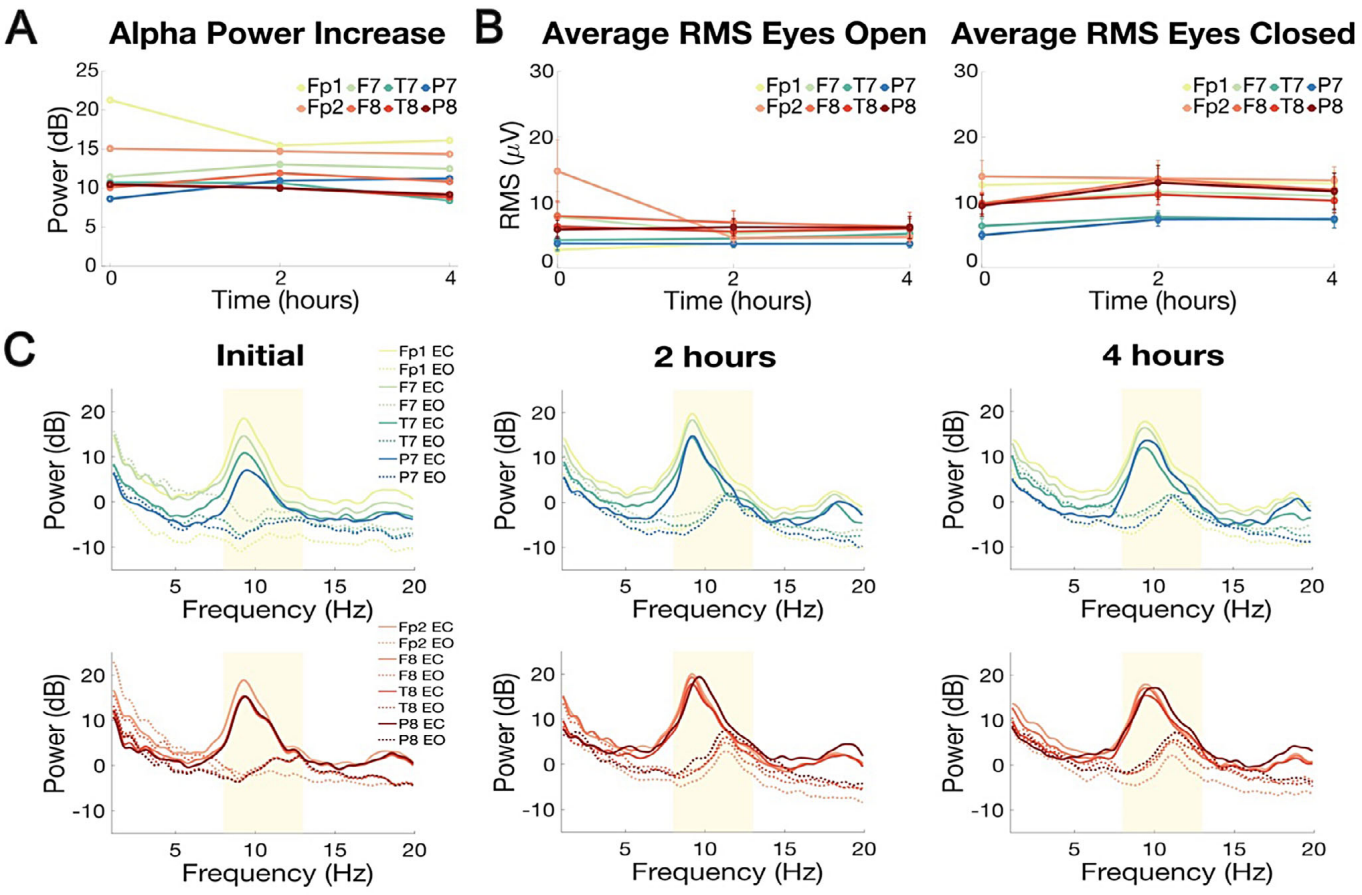
Long‐term EEG using the 8‐channel montage for subject DYOX (data from all other participants are in Figure S9). (A) Difference in alpha‐band power (8‐13 Hz) between eyes‐closed and eyes‐open rest for each electrode across 4 h, showing no significant effect of time (linear mixed‐effects model, α = 0.05; p = 0.45). (B) 2 s window averaged RMS amplitude of the signal on each electrode presented as mean ± SD, separated by resting state condition (left) eyes‐open and (right) eyes‐closed. (C) Power spectral density estimate using 2 s, non‐overlapping windows (dashed lines: eyes‐open, EO; continuous lines: eyes‐closed, EC).

An important aspect of improving EEG is ensuring that recordings can be collected reliably over several hours, enabling high‐fidelity, ecologically valid ambulatory monitoring that is critical for the diagnosis and understanding of epilepsy, psychopathology, and sleep disorders. To assess the long‐term stability of signals collected with our dry‐electrode headband, we recorded repeated 4 min eyes‐open and eyes‐closed blocks across a 4 h session using the same 8‐channel montage. As before, the forehead was cleaned with an alcohol pad, and a few drops of saline were applied to the electrodes only at the start of the session. No additional saline was added thereafter. A clear 10 Hz alpha rhythm peak was present in the eyes‐closed state across all electrodes, with a higher amplitude than in the eyes‐open condition (Figure 7). Throughout the recording session, there was no significant time effect on the mean alpha‐band power difference across the electrodes (linear mixed‐effects model, F(2,21) = 0.83, p = 0.45), and an equivalence test with a smallest effect size of interest of ± 5 dB confirmed no significant changes at 2 h (p_TOST_ = 0.0005, Δ = −0.16 dB, 90% CI [−1.87, 1.54]) and 4 h (p_TOST_ = 0.0008, Δ = −0.84 dB, 90% CI [−2.43, 0.76]) compared to baseline (Figure 8A). Grouped across electrodes in the eyes‐open condition, the mean root mean square (RMS) amplitude changed by −1.650 ± 3.635 µV from 0 to 2 h, and by 0.211 ± 0.487 µV from 2 to 4 h, reflecting minimal drift over time (Figure 8B). We also tested the effects of not adding any saline to the electrodes, and again consistently observed the expected alpha enhancement in eyes‐closed relative to eyes‐open throughout the session, with mean RMS change of 0.855 ± 1.271 µV from 0 to 2 h (Figure S10). Although a mixed‐effects model indicated a statistical time effect on the mean alpha‐band power difference (F(2,21) = 24.89, p = 2.89e‐6), equivalence testing showed the hour‐wise differences compared to initial were meaningfully small (Hour 1: p_TOST_ = 0.0001, Δ = −2.48 dB, 90% CI [−3.13, −1.84]; Hour 2: p_TOST_ = 0.0001, Δ = −2.13 dB, 90% CI [−2.86, −1.41]). These results demonstrate that the addition of saline is not required to preserve physiologic contrast, however its primary benefit is that it substantially improves comfort by softening the sponge interface which facilitates prolonged wear. Together, these findings demonstrate that the dry headband yields reliable high quality multi‐hour EEG suitable for prolonged use.

### 2.7 Wireless Mobile EEG Recordings

**FIGURE 9:**
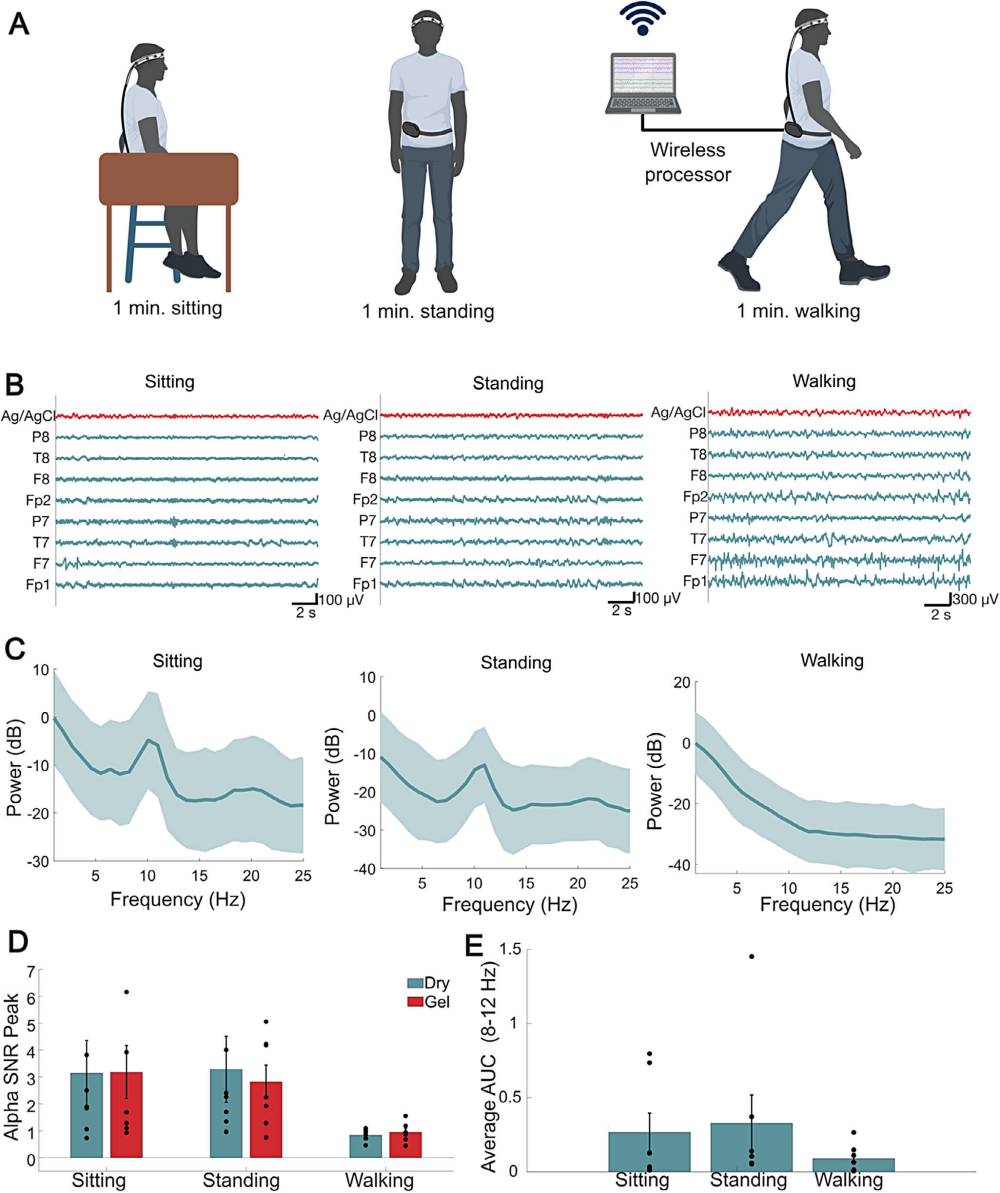
(A) Schematics of the setup and study design for the wireless EEG recordings with the 8‐channel dry Ti_3_C_2_T_x_ electrode headband in ambulatory settings (Created in BioRender. Lab, V. (2026)). (B) Representative time series of broadband EEG band collected with dry Ti_3_C_2_T_x_ and a comparison Ag/AgCl electrode placed near F7 during sitting, standing, and walking (n = 1 participant). (C) Average power spectra of all dry Ti_3_C_2_T_x_ electrodes on the headband (n = 8 electrodes) for a single subject in different states with shaded region representing standard deviation. (D) Average alpha‐band SNR for dry Ti_3_C_2_T_x_ and Ag/AgCl electrodes across all participants (n = 5) in different states (i.e., sitting, standing, and walking) with error bars representing standard error. (E) Average AUC of the PSD from all electrodes on the Ti_3_C_2_T_x_ headset across for each participant (n = 5 participants) in the alpha‐band (8‐12 Hz), showing depression of the alpha rhythm in the walking task with error bars representing standard error.

Another area of increasing clinical and research interest is the use of EEG in remote settings or for ambulatory and at‐home monitoring. Remote and ambulatory EEG require continuous tracking of brain activity with portable EEG units outside controlled clinical environments. Examples of applications include long‐term epilepsy monitoring, early post‐traumatic epilepsy detection, sleep studies, and clinical trials [79, 80], particularly for patients living away from the hospital, in underserved areas, or needing frequent assessments. In ambulatory and at‐home EEG applications requiring unsupervised, reliable, easy‐to‐use EEG systems, gelled electrodes become particularly challenging and impractical. On the other hand, dry electrodes significantly simplify independent set‐up, maintenance, and removal, which ultimately might benefit adoption and compliance [81]. One of the key challenges in developing dry EEG systems for at‐home and ambulatory monitoring is ensuring that the electrodes maintain stable contact with the scalp without shifting and displacements, as even slight movements can introduce motion artifacts that degrade the EEG quality [82]. Here, we sought to investigate the feasibility of using dry Ti_3_C_2_T_x_ electrodes in a portable, wireless mobile EEG system. We captured EEG (n = 5 participants) using the limited montage 8‐channel dry EEG headset and one Ag/AgCl gelled electrode located near F7 and recorded the EEG using a commercial wireless amplifier (Ripple Neuro). The amplifier and wires were bundled and worn in a small, belted bag around the waist for comfort and portability during the study. We acquired the EEG in three different conditions, which were representative of different levels of motion that may occur during ambulatory monitoring: 1) sitting, 2) standing, and 3) walking for up to 2 min at a normal pace (Figure 9A). In all tasks, EEG was acquired in the eyes‐closed resting state condition. During the walking task, participants were able to walk at their own natural walking pace but were guided by study personnel to walk along a straight line for safety. For all the participants, we successfully recorded mobile EEG of a similar quality to gelled electrode (Pearson correlation coefficient, r = 0.85 ± 0.09) and observed a task‐dependent modulation of the alpha rhythm (Figure 9B). Specifically, in standing and sitting states with eyes‐closed, we observed the expected increase in the 8–12 Hz PDR, in relation to the 1/f spectral response, arising in the occipital regions. However, during walking, the PDR was suppressed due to the desynchronization that occurs in the same frequency band during the recruitment of motor cortical regions (Figure 9C) [83, 84]. We quantified and found this task‐dependent PDR suppression with the SNR of the PSD in the alpha range calculated for the three tasks (gel‐sit: 3.18 ± 0.99, dry‐sit: 3.13 ± 1.22, gel‐stand 2.81 ± 0.63, dry‐stand: 3.28 ± 1.23, gel‐walk: 0.94 ± 0.13, dry‐walk: 0.83 ± 0.08) and further verified by the decreasing area under the curve (AUC) value of the PSD in the alpha range during walking (walking: 0.09 ± 0.04, sitting: 0.27 ± 0.13, standing: 0.33 ± 0.19, Figure 9 D,E and Figure S11). Our data indicates that dry Ti_3_C_2_T_x_ electrodes and headset design enable establishing a secure connection with the scalp and recording high‐quality mobile EEG.

## 3 Conclusions

In this study, we advanced and validated a soft, low‐impedance, TMS‐compatible dry EEG electrode technology based on Ti_3_C_2_T_x_ MXene, with superior usability, versatility, inclusivity, and comfort than conventional wet and dry EEG electrodes. To address the issues with fabrication complexity and cost that limit many of the current dry electrode systems and to allow full customizability of the electrode geometry and EEG setup, we propose a rapid, scalable, and high‐throughput process based on dip‐coating porous and compressible PVA templates in aqueous Ti_3_C_2_T_x_ suspensions. The ease of manufacturing allows for the rapid electrode prototyping and fabrication, and scale‐up to mass manufacturing, even in limited resource settings. The electrode fabrication method can be extended to other 2D nanomaterials as well, so long as they are colloidally stable in aqueous dispersions and allow for solution‐based processing. Dry Ti_3_C_2_T_x_ electrodes have low impedance with minimal skin prep and no gel at levels comparable to clinical‐grade gelled electrodes (<10 kΩ at 10 Hz) and significantly lower than conventional dry electrodes, including metal and polymer‐based devices (>50 kΩ) [20]. Dry Ti_3_C_2_T_x_ electrodes also maintain stable contact with the scalp without the application of high mechanical pressure. This leads to a more comfortable experience for end users without compromising signal quality. Furthermore, the Ti_3_C_2_T_x_ electrodes show excellent electrochemical and mechanical stability to repeated compression cycles, which are crucial to ensure comfort, durability, and quality of EEG recordings in long‐term monitoring and repeated use where the electrodes may be repeatedly compressed against the scalp.

Dry Ti_3_C_2_T_x_ electrodes do not show mechanical displacement, heating, or harmful charge at the surface when they are placed under the TMS coils and interact with fields commonly adopted for neurological therapy and research. Added to the MRI compatibility of Ti_3_C_2_T_x_ [51], the TMS‐compatibility of dry Ti_3_C_2_T_x_ electrodes significantly reduces the risk of motion artifacts and electrical interference during concurrent TMS‐EEG. These artifacts and EEG signal contamination have been a persistent challenge in previous TMS‐EEG studies [85]. The ability to manufacture low‐profile electrodes reduces the required TMS machine output to surpass cortical thresholds, decreasing hardware wear‐and‐tear and making TMS‐EEG possible for individuals with lower cortical excitability [15]. Moreover, the minimal phase distortions evident in MXene electrodes support their use in real‐time TMS‐EEG applications [86, 87, 88].

Taking advantage of the versatility of the fabrication process, we integrated the dry Ti_3_C_2_T_x_ electrodes into custom‐designed multichannel headsets for both full‐scalp and reduced montage EEG, replicating standard configurations that are currently adopted for both clinical and research use and validated the performance across different environments, setups, and commercial acquisition systems (Table S2). The headsets provide a comfortable and adjustable fit, accommodating various head sizes and hair types, ensuring inclusivity in clinical and research applications (Figure S12). This is particularly beneficial as traditional gelled EEG systems at present undergo great difficulty in maintaining contact with the scalp in populations with dense hair types, leading to inadequate data collection that affects diagnostics and perpetuates disparities in neurological care and research [13, 89, 90]. The ability to interface with clinical systems is crucial in ensuring that new technology advances can be translated to clinical environments and are accessible to all demographics. The predefined electrode locations and ease of electrode placement and removal are added advantages compared to clinical systems where electrodes must be placed one at a time by expert technicians. Additionally, Ti_3_C_2_T_x_ MXene electrodes can be employed with minimal to no skin preparation, further cutting down on application time. The cap can be removed from the participant's scalp without leaving residues or material that needs to be washed off, as is typical with gelled systems that often deter participation in EEG studies.

Our studies confirm that the Ti_3_C_2_T_x_ MXene electrodes can reliably capture EEG signals comparable to traditional gel‐based Ag/AgCl electrodes in different static, mobile, and long‐term EEG use case scenarios, with both full and limited montage configurations and even without any prior skin preparation. Additionally, we demonstrate the potential for ambulatory and wireless EEG monitoring, with the electrodes maintaining reliable signals during dynamic tasks such as walking. This is particularly important for applications requiring continuous monitoring in real‐world settings, where motion artifacts and environmental noise can significantly degrade EEG quality and diagnostic information. By maintaining signal integrity during movement, our dry MXene‐based system addresses a major limitation of traditional gel‐based EEG, which often struggles in ambulatory conditions due to signal disruption due to several factors (shearing potential, gel drying, movement of leads, etc.) [91]. Our technology could be effectively used in remote settings or for continuous monitoring in everyday environments, providing opportunities for reliable monitoring and management of neurological disorders, expanding access, and ultimately improving quality of care. Such capabilities are especially advantageous for conditions such as epilepsy, where capturing transient events during daily activities is crucial, or for sleep studies conducted outside of controlled laboratory environments.

Our electrode design and materials also offer benefits in terms of environmental impact. In contrast to gel Ag/AgCl and dry metal electrodes, which rely on substantial amounts of silver, gold, or stainless steel and generate persistent e‐waste, our design minimizes metal content and uses a PVA substrate that can be found in biodegradable systems. Additionally, the degradation product of MXene in TiO_2,_ is an environmentally safe material commonly used in the cosmetic and food industries. Finally, MXene/PVA electrodes are mechanically robust and could be reused multiple times, reducing the number of disposable electrodes required per recording session.

In conclusion, the development of dry Ti_3_C_2_T_x_ electrodes represents an advance in EEG technology and a viable solution for comfortable low‐impedance EEG monitoring in real‐world applications and on different demographics and hair types. These electrodes offer a comfortable, versatile, and scalable solution for both clinical and research applications, addressing many of the limitations associated with traditional gel‐based and dry electrodes.

## 4 Methods and Materials

### 4.1 Fabrication of Dry Ti_3_C_2_T_x_ MXene Electrodes

Circular discs were cut out of a sheet of hydroxylated poly‐vinyl acetate (PVA, Medtronic USA) with an 8 mm biopsy punch. Each disc was then dip‐coated in an aqueous dispersion of Ti_3_C_2_T_x_ MXene (20 mg mL^−1^, Murata Manufacturing Inc.) and then dried in air. Subsequently, an Ag/AgCl button snap connector was attached to one side of the Ti_3_C_2_T_x_‐PVA sponges with conductive silver epoxy. The epoxy was cured in an oven at 80°C.

### 4.2 Scanning Electron Microscopy

Scanning electron microscope (SEM) images were collected on the FEI Quanta 600 FEG Mark II Environmental SEM in high vacuum (5×10^−5^ Torr), with the backscattered electron signals and energy‐dispersive X‐ray spectrometry (EDS) detector installed for elemental analysis. The Ti_3_C_2_T_x_‐PVA samples were imaged with a working distance of 9.3 mm, overall electron beam acceleration of 10 kV, and a spot size of 6. For the raw PVA sample, the working distance was 8.9 mm. SEM images of the Ti_3_C_2_T_x_‐PVA were acquired with a 30 µs dwell time, integrating 4 frames, while the PVA sample was integrated with 16 frames to prevent drifting.

### 4.3 Raman Spectroscopy Analysis

Raman spectroscopy was performed with a Horiba Raman and AFM system with a 785 nm excitation laser (50x objective, 25% ND filter). Raman spectra of Ti_3_C_2_T_x_‐PVA were acquired for 30 s per scan and averaged across 5 scans, while Raman spectra of uncoated PVA were acquired for 20 s per scan and averaged across 5 scans.

### 4.4 Headset Fabrication

The EEG headset is composed of elastic fabric (69% polyester, 31% rubber), which provides flexibility and comfort while supporting electrode placement. Using the regions marked on the EEG 10–20 system, 8 mm diameter holes were cut out for 8 electrodes in positions Fp1, F7, T3, T5, Fp2, F8, T4, and T6 and 21 electrodes for the full scalp coverage cap (electrode locations spaced approximately 5 to 6 cm apart). To secure the headband across varying head sizes, a plastic clasp is sewn onto one end of the headband, with additional elastic material added to the end of the band to adjust according to the participant's head size. Snap‐in holders for snap‐on leads (Shimmer, Biophysical Lead Pack) that interface with the electrodes were designed using SolidWorks and 3D printed in Formlabs Durable Resin on a Form3 SLA 3D printer. The leads were modified with touchproof terminations to interface with a variety of different EEG acquisition systems (1.5 mm touchproof connectors).

### 4.5 Impedance Testing

Electrochemical impedance spectroscopy (EIS) measurements on the Ti_3_C_2_T_x_‐PVA, 20 mm disk and 3 mm cup Ag/AgCl electrodes (n = 5 electrodes of each type) were collected with a Gamry Reference 600 potentiostat at room temperature on a conductive agarose phantom (0.6% w/w agarose and 0.3% w/w NaCl) in a three‐electrode configuration. Two Ag/AgCl (20 mm pre‐gelled disk or 3 mm gelled cup) electrodes were used as reference and counter electrodes. Impedance and phase spectra were collected in the 1 Hz–1 MHz frequency range with a 10 mV_rms_ input AC voltage. Impedance was also collected on 5 participants using the Ti_3_C_2_T_x_‐PVA electrodes (n = 10 electrodes), with 20 mm disk Ag/AgCl electrodes used as reference and counter electrodes (placed on the mastoid). Impedance and phase spectra were again collected in the 1 Hz–1 MHz frequency range with a 10 mV_rms_ input AC voltage. For equivalent circuit modeling, EIS measurements were also performed in a standard three‐electrode electrochemical cell using a single‐frit Ag/AgCl reference electrode, a large graphite rod counter electrode, and an 8 mm Ti_3_C_2_T*_x_* MXene electrode as the working electrode. Electrochemical measurements were collected from 100 kHz to 1 Hz on a conductive agarose phantom.

### 4.6 Conductivity Measures

Rectangular sponge samples (n = 4, 1.15 × 0.70 × 0.50 cm) were immersed in a 20 mg mL^−1^ MXene ink dispersion for 5 min and air‐dried before electrical testing. Conductivity was measured with a collinear four‐point probe in two orthogonal orientations to assess anisotropy. For each orientation, three sequential readings were acquired at four locations along the specimen and reduced to a single value by taking the median to suppress contact‐settling outliers. When the thickness of the material is much higher than the space between the probes, bulk resistance due to spherical protrusion of current emanating from the outer probe tips can be calculated as

(1) $$\rho =2\pi \;s\left(\frac{V}{I}\right)F$$

where *s* is the uniform probe spacing, t is the specimen thickness, and *F* is the correction factor d/s for a rectangular sample of width *d*. Because our geometry satisfied d/s >> 1, we can approximate the correction factor to be 1. Conductivity was then calculated as:

(2) $$\sigma =\frac{1}{\rho }$$

### 4.7 Mechanical Testing

Ti_3_C_2_T_x_‐PVA samples (n = 5) were compressed to 80% of their height on an Instron tensile tester using a 15 N load cell for up to 50 cycles. The resulting force and extension were measured. The load and extension for each cycle of compression and decompression were plotted against each other, and the difference in area between the resulting two curves for each cycle was calculated as the hysteresis loss. After each compression, impedance was recorded with EIS as described above.

### 4.8 Statistical Analysis

Impedance at 10 Hz was compared between the initial measurement (cycle 0) and the final measurement (cycle 5) across electrodes using a two‐tailed Mann‐Whitney U test with significance defined as p ≤ 0.05. Data was analyzed and plotted with MATLAB.

### 4.9 TMS Safety Testing

#### 4.9.1 TMS System

TMS pulses were delivered by a Magstim SuperRapid 2 unit paired with a 3910‐00 AFC coil. The electrodes were connected directly to the jackbox of the amplifier system and the Bittium EXG/LP hardware filters were set on a Bittium NeurOne system as recommended for TMS studies. All signal recordings were conducted at a sampling rate of 10 kHz and in DC mode to ensure compatibility with TMS protocols.

#### 4.9.2 TMS Protocols

For the displacement experiments, we tested two different TMS protocols consistent with the upper bound of machine output required to meet excitability thresholds across individuals: 10 Hz repetitive TMS (rTMS) at 80% machine output for 10 s, and 50 Hz theta burst stimulation at 45% machine output for 10 s. Additionally, single pulses were delivered at 60%, 70%, 80%, and 90% machine output (n = 1 per stimulation condition). For temperature and induced charge measurements trials (n = 2 and n = 3, respectively), the same TMS protocols were applied, consisting of 10 Hz stimulation at 80% output and 50 Hz theta burst at 45% output, for 30 and 40 s intervals, respectively.

#### 4.9.3 Experimental Setup

For displacement testing, electrodes were suspended in air at 1 mm from the TMS coil, eliminating direct mass interaction and minimizing unwanted electrical bridging, while allowing for the detection of any potential movement during stimulation. Electrode displacement was recorded at 30 frames‐per‐second using a Canon EOS M50 high‐definition digital camera, capturing real‐time video of the electrodes during TMS pulses. The videos were analyzed with custom MATLAB code, allowing for object selection and quantification of displacement. For temperature and induced voltage/charge tests, the electrodes were mounted on a conductive phantom (0.6% w/v agarose, 0.17% w/v NaCl), which simulates the mechanical and electrical properties of human tissue [66, 92, 93, 94, 95]. Fiber optic temperature probes (PRB 500, Osensa: ‐40 to 100 °C detection range, ± 0.10°C accuracy) were placed between the electrodes and the agarose phantom to monitor the temperature before, during, and after TMS. Induced voltage across electrode pairs was measured with a Tektronix TBS 2000 oscilloscope, and the OpenChoice desktop (Tektronix) software was used for data acquisition. The resulting charge density σ was calculated by integrating the current, *I*, recorded at the electrode generated by a TMS pulse of duration *t*, divided by the geometric area of the electrode *A* according to the following equation:

(3) $$\sigma =\frac{1}{A}\int_{o}^{\Delta t}I\left(t\right)dt$$

Data was analyzed and plotted with MATLAB.

### 4.10 Full Scalp Dry EEG Recordings

#### 4.10.1 EEG Acquisition

Full‐scalp EEG recordings were acquired on healthy volunteers (n = 5) at Drexel University with the 21‐channel dry Ti_3_C_2_T_x_ EEG headset (10‐20 montage). The study was reviewed and approved by the IRB of Drexel University (protocol no. 1904007140), and informed consent was obtained from all participants. On each participant, the dry EEG headset was placed on the scalp using the nasion as an anatomical landmark for alignment. Then, the scalp under each dry electrode was wiped with a 70% alcohol pad, followed by the addition of a drop of ∼1 mL of 1× phosphate buffered saline (PBS). Three passive, gelled Ag/AgCl cup electrodes (Technomed Disposable EEG Cup Electrodes) were also placed adjacent to dry electrodes on Fpz, C3, and Oz for comparison. The scalp area under each Ag/AgCl cup electrode was first prepped using an alcohol wipe and abrasive wipe, followed by gel application to interface the cup electrode. Additional gelled Ag/AgCl cup electrodes were placed on the right and left mastoid and used for ground and reference, respectively. All dry Ti_3_C_2_T_x_, gelled Ag/AgCl, ground, and reference electrodes were simultaneously connected to the jack box of a Bittium NeurOne Tesla EEG system (Bittium Corporation, Finland). EEG was acquired in a shielded room at a sampling rate of 5 kHz. We recorded EEG while participants performed a 2 min resting‐state block with their eyes open. In this block, participants sat quietly and looked at a fixation cross on a computer screen. We then recorded EEG during a 2 min block of a 20 Hz steady‐state visually evoked potential (SSVEP) paradigm. We used PsychoPy [96] to display a basic luminance‐based SSVEP paradigm where a square in the center of the computer screen flickered between black and white at 20 Hz on a grey background. Due to the system load, the actual flickering rate presented to the participants varied between 20.3 Hz and 20.9 Hz, measured by the software. The flickering rate was consistent within each participant, and these small fluctuations did not impact the analysis other than resulting in slight shifts of the SSVEP peak response in the EEG spectrum.

#### 4.10.2 Data Analysis

We used MATLAB R2024b update 4 (Mathworks, Inc., Natick, Massachusetts, USA), EEGLAB [97], ERPLAB [98], and custom functions to preprocess and analyze the data. We converted the data to EEGLAB structures and individually ran each participant's data through a semi‐automatic preprocessing pipeline. We used a non‐causal FIR filter to bandpass filter the data from 1–50 Hz and notch filter the data at 25 Hz to remove a known building‐related environmental noise artifact. Both filters had transition bandwidths of 1 Hz and cutoff frequencies (−6 dB) of 0.5, 50, and 24.5, 25.5, respectively (function ‘pop_eegfiltnew’) [99]. We then used the function *eeg_regepochs’* to add event markers to our data every 5 s and the function *‘pop_clean_rawdata’* to remove artifacts. We used the default settings in *‘pop_clean_rawdata’*, did not include channel rejection, and used artifact subspace reconstruction instead of deleting noisy data segments. After cleaning, we re‐referenced the data to Fp2 [100]. We then used ERPLab to epoch the data into 5 s epochs without baseline correction and compute the average ERP total power spectrum. The resulting output was the spectral power by frequency and channel of the averaged 5 s ERPs. We then calculated a neighbor‐based signal‐to‐noise ratio (SNR) where the SNR is the ratio of PSD at a given frequency to the mean PSD of its neighbors. This SNR approach is less sensitive to high spectral power density and more sensitive to rapid increases in spectral power density, making it ideal for the sharp, narrow peaks in spectral power density associated with SSVEPs [40, 101]. In our study, the frequency resolution was 0.1526 Hz, and we included the five points before and the five points after the frequency of interest in the mean PSD.

#### 4.10.3 Statistical Analysis

To quantify the SSVEP effect, we extracted the 20 Hz SNR for each participant by averaging across the occipital/parietal region of interest channels (P3, P4, Pz, O1, O2) separately for eyes‐open rest and visual stimulation blocks. Condition differences were assessed using a two‐tailed paired t‐test across all participants, with significance defined as p ≤ 0.05.

### 4.11 EEG Recordings With 8‐channel Dry Ti_3_C_2_T_x_ Headband

All EEG studies described in this section were approved by the Institutional Review Board (IRB) of the University of Pennsylvania (protocol no. 832421).

#### 4.11.1 Clinical EEG

Patients who were already scheduled to receive a routine EEG in the outpatient EEG clinic of the Hospital of the University of Pennsylvania (HUP) were recruited to take part in this study, which took place ∼30 min prior to their clinical exam (n = 5 participants). The 8‐channel dry Ti_3_C_2_T_x_ headband was placed on the participant with the front tab centered on the nasion landmark and then adjusted to their head size using the clasp at the back of the band. Two 3 mm Ag/AgCl Natus gelled cup electrodes were placed on the midline for reference and ground for clinical recordings. The skin under the gelled electrode was prepped using an abrasive paste and conductive paste. Then, the gelled electrode was placed on the scalp and secured with adhesive tape. The skin under each dry electrode contact was wiped with a 70% alcohol preparation pad, followed by the addition of a drop of approximately 1 mL of 1X PBS.

The Natus EEG amplifier system and software were used to record EEG (250 Hz sampling rate). Participants were asked to open and close their eyes for 2 to 3 min durations during the recording, 4 to 5 times while lying down. The dry electrode cap was removed at the end of the session and clinical Ag/AgCl Natus gelled electrodes were placed on the full scalp in locations corresponding to the 10–20 system, in addition to the locations used for MXene recording for the routine EEG, in which the same eyes open and close tasks were completed for the same intervals and repetitions while the participant remained laying down in the same position that they were in for the dry electrode recording. The same Ag/AgCl reference and ground electrodes placed on the midline for the dry electrodes recording were kept in place and used for reference and ground in the clinical recording. All data was de‐identified and stored on ieeg.org. All data recorded (5 dry electrodes and 5 gelled electrode recordings were then reviewed by a board‐certified neurologist on the Natus software to check for readability and ensure no major artifacts or signal interruptions were present.

#### 4.11.2 Data Analysis

Python toolboxes (numpy, matplotlib, and scipy) and custom functions were used to preprocess and analyze the data. Only the annotated section of data corresponding to ‘eyes‐closed’ task was used for analysis. The data for each participant was first re‐montaged to a bipolar montage and then low pass filtered using a fourth order Butterworth filter at a cut off frequency of 50 Hz. The power of each signal was computed using a Welch transform and plotted against respective frequencies. Spectrograms were generated using the *‘specgram’* function.

#### 4.11.3 Long‐Term EEG Recordings

Repeated resting state EEG during prolonged wear was collected with the 8‐channel dry Ti_3_C_2_T_x_ headband on healthy volunteers (n = 6) at Drexel University (protocol no. 1904007140). The forehead was cleaned with a 70% alcohol pad, and the headband was positioned using the nasion for alignment. For five participants, a small amount of saline was applied once to the electrode‐skin interface at the start of the session and was not readministered thereafter during the entire session. One participant completed the entire protocol without any saline or scalp preparation at any point during the session. In all cases, gelled Ag/AgCl cup electrodes were placed on the right and left mastoids (standard skin prep) and used as ground and reference, respectively. Data were acquired on the Bittium NeurOne Tesla EEG system at 500 Hz sampling rate across all blocks. EEG was recorded while participants performed alternating 4 min resting‐state blocks with their eyes open and then eyes closed. This sequence was conducted every hour for 4 h, with the headband continuously worn in between recordings. Two datasets were excluded due to bad channels that precluded reliable PSD and RMS estimation, leaving 4 participants for analysis.

#### 4.11.4 Data Analysis

All analyses were performed in MATLAB R2024b. Preprocessing used EEGLAB to band‐pass filter data from 1–30 Hz and remove blink artifact‐related components from each channel in the eyes‐open recordings via FastICA. For each trial, the first and last 10 s were discarded to avoid filter transients, yielding 220 s segments. Each channel was segmented into non‐overlapping 2 s windows, and power spectral density was computed using Welch's method *‘pwelch’*. Per‐channel alpha‐band power was obtained from the window‐averaged PSD in the 8–13 Hz region using the function *‘bandpower’*. The alpha‐band power difference was defined as the eyes‐closed minus eyes‐open value. For signal stability, RMS amplitude was computed within each 2 s window and averaged across windows to obtain per‐channel means and standard deviations for both eyes‐closed and eyes‐open conditions at each hour.

#### 4.11.5 Statistical Analysis

Time effects on the signal were tested with a linear mixed‐effects model including a fixed effect of hour and a random intercept for electrode (*‘fitlme’* and ‘*anova’*). Significance was set at p ≤ 0.05. We also performed a two one‐sided t‐test to compare the alpha‐band power difference at each hour to baseline (hour 0) per electrode, using a smallest effect size of interest of ±5 dB and 90% confidence (‘*tcdf’* and ‘*tinv’*).

#### 4.11.6 Mobile EEG

EEG was acquired from healthy participants at the University of Pennsylvania, and informed consent was obtained from all participants (n = 5). The dry Ti_3_C_2_T_x_ headband placement and skin prep procedures were the same as described in the Methods for Clinical EEG recordings, with an additional 20 mm Ag/AgCl Natus pre‐gelled disk electrode placed on the forehead next to F8 for comparison. In this ambulatory study, we connected the dry and Ag/AgCl electrodes to a portable amplifier (RippleNeuro Grapevine) and collected EEG wirelessly at 30 kHz sampling rate (default for wireless recording) on a laptop, where the data were locally stored and deidentified, using RippleNeuro Trellis acquisition software. The amplifier was placed in a small belt bag and worn around the participant's waist for the duration of the experiment. Participants were asked to sit in a chair, then stand in place, and finally walk at a natural pace with their eyes closed for 1 min each. During the walking portion, the participants were instructed to walk at their own pace but were hand guided by lab personnel to walk along a straight line for the duration of the recording to ensure they were able to move safely.

#### 4.11.7 Data Analysis

All data was processed and analyzed using MATLAB 2022b. Segments from the start and end of the recording were clipped to exclude periods where connectors were being adjusted or while participants were transitioning to tasks. For consistency, the same length was maintained across all tasks per subject, and a minimum of 30 s was included in the analysis. The signals were bandpass filtered using a second order Butterworth filter (*‘butter’* for filter creation and *‘filtfilt’* with cutoff frequencies ranging from 0.5–100 Hz, encompassing all relevant frequency bands. Similarity between dry and gelled EEG signals was quantified using the Pearson correlation coefficient computed on the filtered time‐domain recordings within each task for each participant. Values are expressed as mean ± SD. The PSD was computed for all electrode channels using Welch's power spectral density estimate, implemented through the *‘pwelch’* function in MATLAB. To quantify the task‐dependent depression of the alpha rhythm (8‐12 Hz), the area under the curve (AUC) in this frequency range was computed and compared across all tasks. The PSD across all eight dry channels was averaged to generate a single curve for each task. These curves were subsequently normalized by dividing by their maximum. Subsequently, the *‘cumtrapz’* function was employed over the 8–12 Hz range to compute the cumulative trapezoidal summation, representing the discrete integral across the alpha‐band. The average AUC in the alpha frequency band and standard error was calculated across all subjects. The PSD was calculated for each electrode to quantify their respective SNR, which was calculated using established methods [102]. The SNR value of the alpha peak was identified per task for each electrode type using the ‘*findpeaks’* function.

## Funding

This work was supported by the National Institutes of Health (NIH) grant no. R01NS121219 (F.V., J.D.M., and K.A.D.) and Starfish Neuroscience, Inc. (J.D.M).

## Conflicts of Interest

F.V. is a co‐inventor on the US patent. US11925466B2 â€œImplantable devices using 2D metal carbides and nitrides (MXenes).

## Supporting information




**Supporting File**: advs74257‐sup‐0001‐SuppMat.pdf.

## Data Availability

Data will be made available upon reasonable request.
