# Supplementary material for: Advancing Dry Electroencephalography With Scalable, Soft, and Transcranial Magnetic Stimulation‐Compatible Ti3C2Tx MXene Electrodes for Research and Clinical‐Grade Applications
Source: Adv Sci (Weinh). 2026 Feb 15;13(21):e11486. doi: 10.1002/advs.202511486 (PMC13073255; doi:10.1002/advs.202511486)
Supplement: Supplementary file 1 — Supporting File: advs74257‐sup‐0001‐SuppMat.pdf. [file ADVS-13-e11486-s001.pdf]

**Supplementary Information**

Sneha Shankar[1,2,3], Jakob Michiels[1,2,3*], Ksenija Tasich[1,2*], Ashley Koluda[1,2], Ryan Rich[4], Brian Erickson[4], Eugenia Angelopoulos[2], Francesca Cimino[1,2], Daryl Hurwitz[1,2], Raghav Garg[2,3,5], Spencer R. Averbeck[1,2,3], Doris Xu[1,2], Mariam Josyula[2,5], Nina Petillo[2,5], James J. Gugger[2,5,6], Kathryn A. Davis[2,5¶], John Medaglia[4,5,7¶], Flavia Vitale[1,2,3,5,8]

[1] *Department of Bioengineering, University of Pennsylvania, Philadelphia, PA, United States*

[2] *Center for Neuroengineering & Therapeutics, University of Pennsylvania, Philadelphia, PA, United States*

[3] *Center for Neurotrauma, Neurodegeneration, and Restoration, Corporal Michael J. Crescenz Veterans Affairs*

[4] *Applied Cognitive and Brain Sciences, Department of Psychological & Brain Sciences, Drexel University, Philadelphia, PA, United States*

[5] *Department of Neurology, University of Pennsylvania, Philadelphia, PA, United States*

[6] *Department of Neurology, University of Rochester, Rochester, NY, United States of America*

[7] *Department of Neurology, Drexel University, Philadelphia, PA, United States of America*

[8] *Department of Physical Medicine & Rehabilitation, University of Pennsylvania, Philadelphia, PA, United States*

*Corresponding Authors E-mail: vitalef@pennmedicine.upenn.edu*

[*, ¶] *These authors contributed equally.*

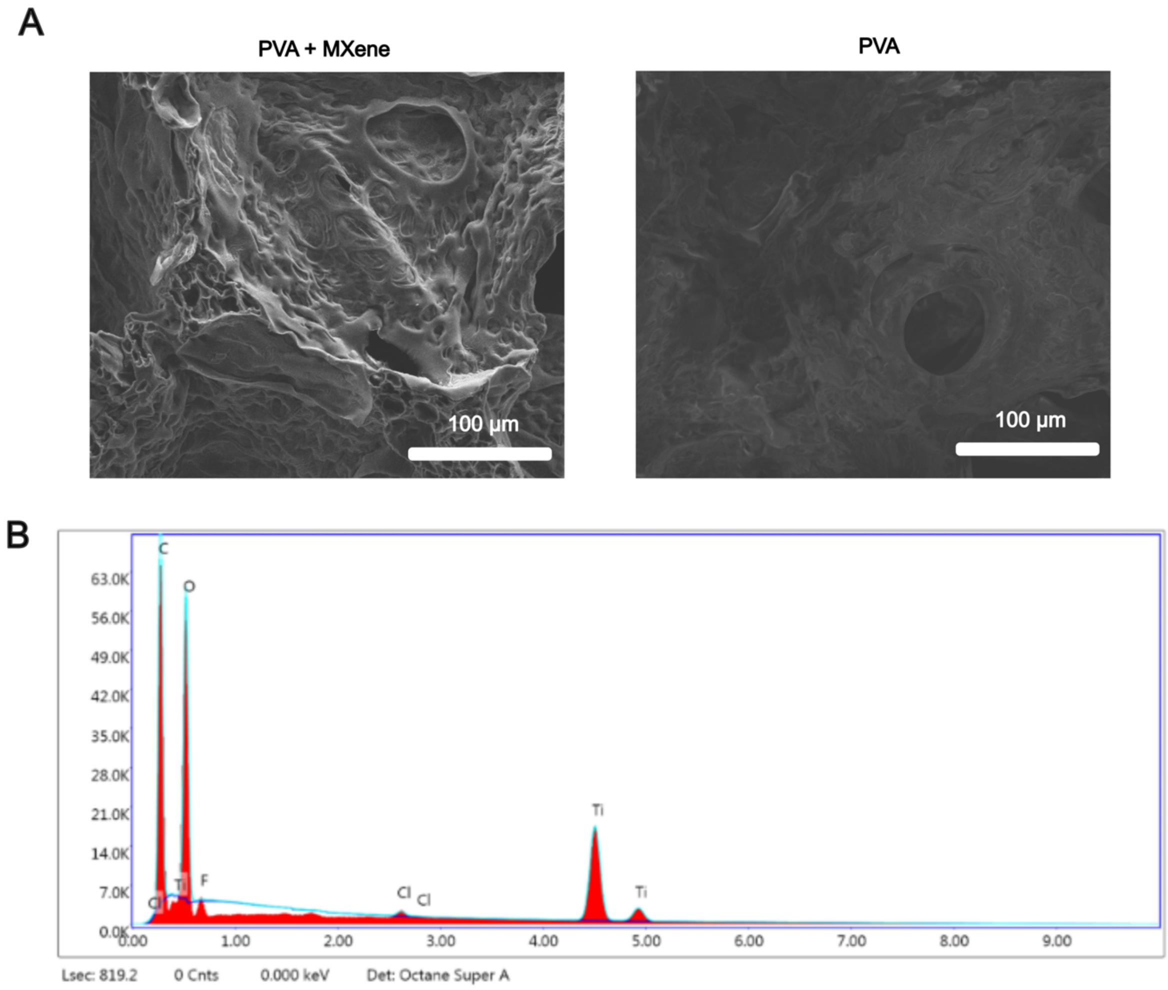


| Element | Weight % | Atomic % | Net Int. | Error % | Kratio | Z | A | F |
|---|---|---|---|---|---|---|---|---|
| C K | 21.13 | 39.45 | 433.20 | 4.10 | 0.1801 | 1.0838 | 0.7862 | 1.0000 |
| O K | 25.07 | 35.14 | 391.30 | 7.89 | 0.1084 | 1.0329 | 0.4184 | 1.0000 |
| F K | 0.16 | 0.19 | 2.90 | 29.57 | 0.0007 | 0.9588 | 0.4641 | 1.0000 |
| ClK | 0.74 | 0.47 | 10.00 | 6.70 | 0.0065 | 0.8744 | 0.9931 | 1.0210 |
| TiK | 52.89 | 24.76 | 252.10 | 3.75 | 0.4301 | 0.8071 | 1.0044 | 1.0030 |

**Supplementary Figure S1**. (A) SEM images of PVA discs before and after absorption of the $Ti_3C_2T_x$ aqueous dispersion (~700 X magnification). (B) EDX spectrum of inked $Ti_3C_2T_x$ + PVA pillars.

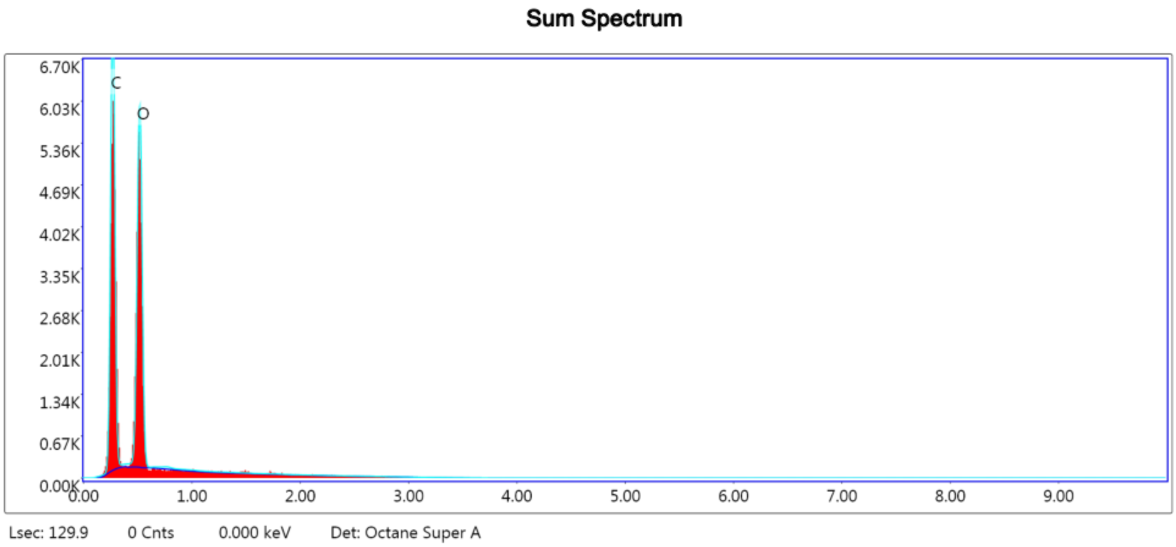


**eZAF Smart Quant Results**

| Element | Weight % | Atomic % | Net Int. | Error % | Kratio | Z | A | F |
|---|---|---|---|---|---|---|---|---|
| C K | 52.93 | 59.96 | 289.10 | 3.10 | 0.4726 | 0.9610 | 0.9293 | 1.0000 |
| O K | 47.07 | 40.04 | 254.80 | 6.10 | 0.2773 | 0.9129 | 0.6453 | 1.0000 |

**Supplementary Figure S2**. EDX spectrum of uncoated PVA pillars.

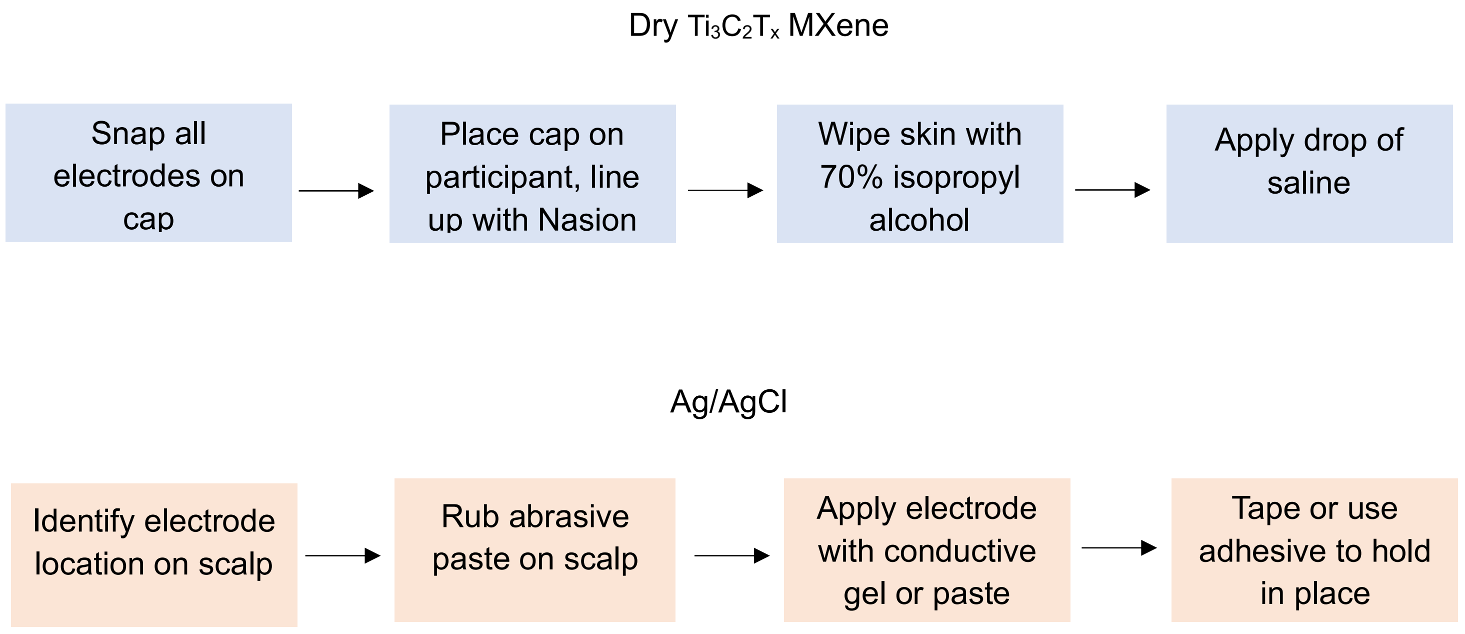


**Supplementary Figure S3**. Skin preparation protocol for (top): dry $Ti_3C_2T_x$ MXene and (bottom): gelled electrodes

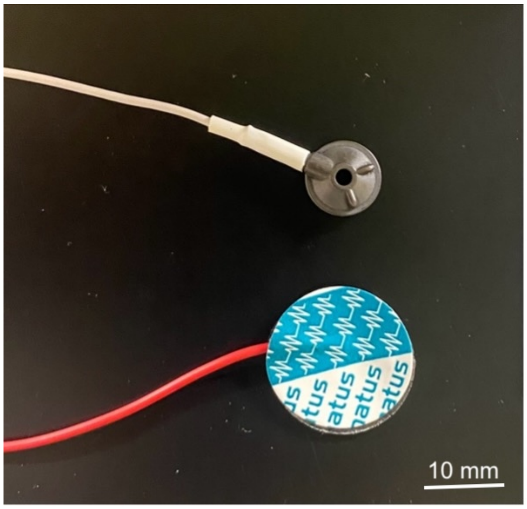


**Supplementary Figure S4**. Ag/AgCl electrodes used for the 4.5 impedance tests in agarose phantoms: (top) 3 mm gelled cup electrode (10 mm diameter), (bottom) gelled disc electrode (20 mm diameter).

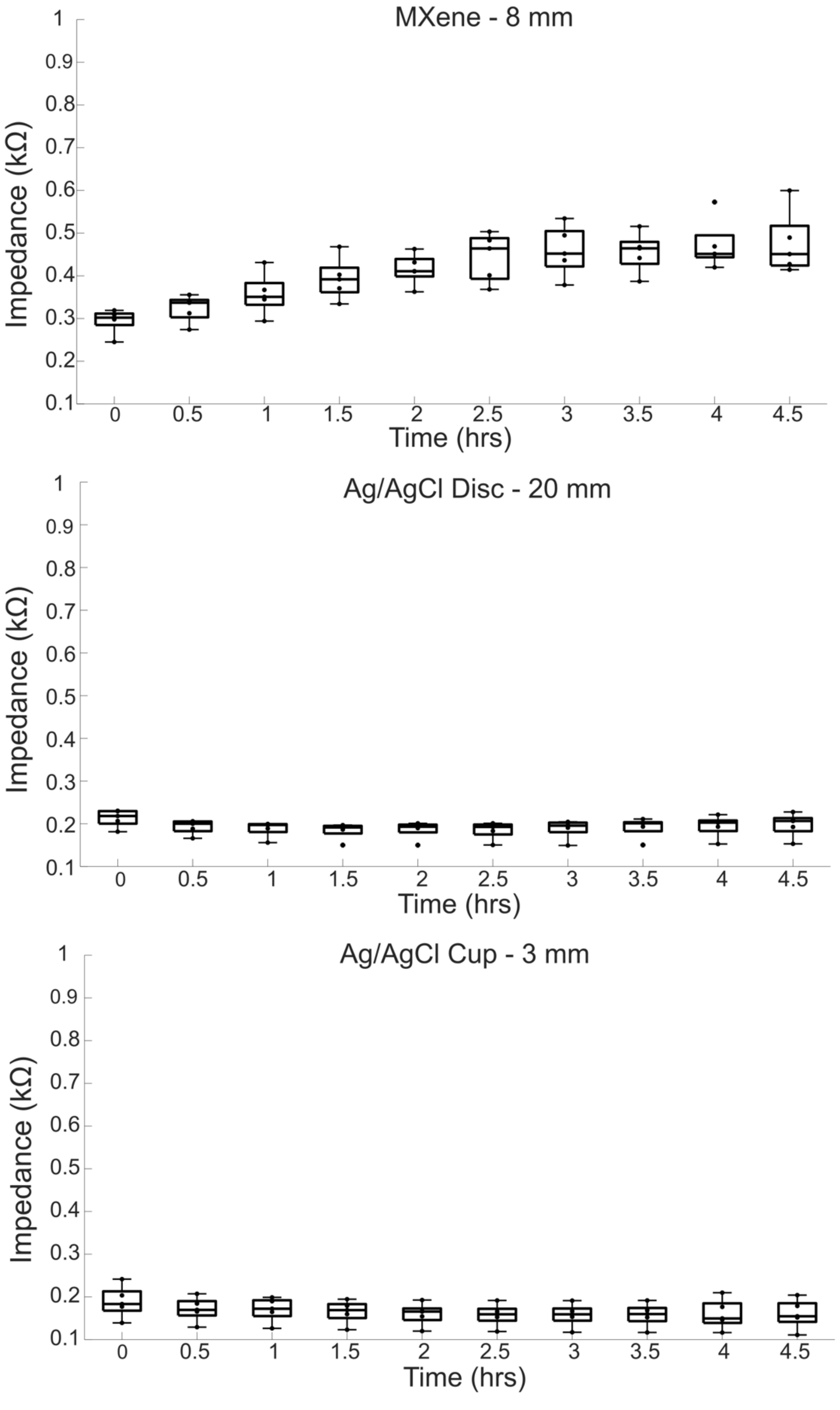


**Supplementary Figure S5**. Average 10 Hz impedance over 4.5 hours on agarose phantom for dry $Ti_3C_2T_x$ and gelled Ag/AgCl (disc and cup) electrodes (n=5 electrodes of each type).

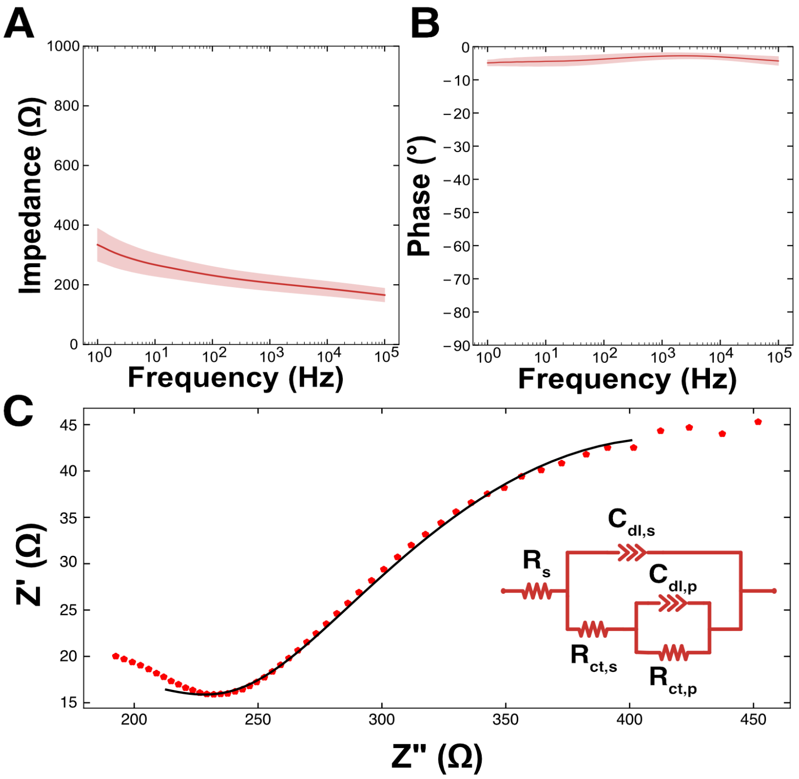


**Supplementary Figure S6. Impedance spectroscopy and equivalent circuit model fitting. (A,B)** Broadband (**A**) impedance and (**B**) phase magnitude of 8 mm $Ti_3C_2T_x$ MXene electrodes. Traces show mean ± one standard deviation (n = 10). (**C**) Nyquist profile and fitting of a representative electrode. Inset shows the equivalent circuit model of porous $Ti_3C_2T_x$ MXene electrodes. Raw data as red stars, model fit as black line.

**Supplementary Table S1.** Equivalent circuit model parameters and values.

| Circuit Parameter | Mean | Standard Deviation |
|---|---|---|
| $R_s$ (Ω) | 102.4 | 40.2 |
| $R_{ct,\ surface}$ (Ω) | 195.5 | 40.8 |
| $C_{dl,\ surface}$ (nF $cm^{-2}$) | 19.9 | 17.8 |
| $R_{ct,\ porous}$ (Ω) | 144.9 | 68.3 |
| $C_{dl,\ porous}$ (μF $cm^{-2}$) | 552.4 | 383.7 |
| $R_{ct}$* (Ω) | 340.4 | 69.5 |
| $C_{dl}$* (μF $cm^{-2}$) | 552.4 | 383.7 |

*$R_{ct} = R_{ct,\ surface} + R_{ct,\ porous}$ in series, *$C_{dl} = C_{dl,\ surface} + C_{dl,\ porous}$ in parallel

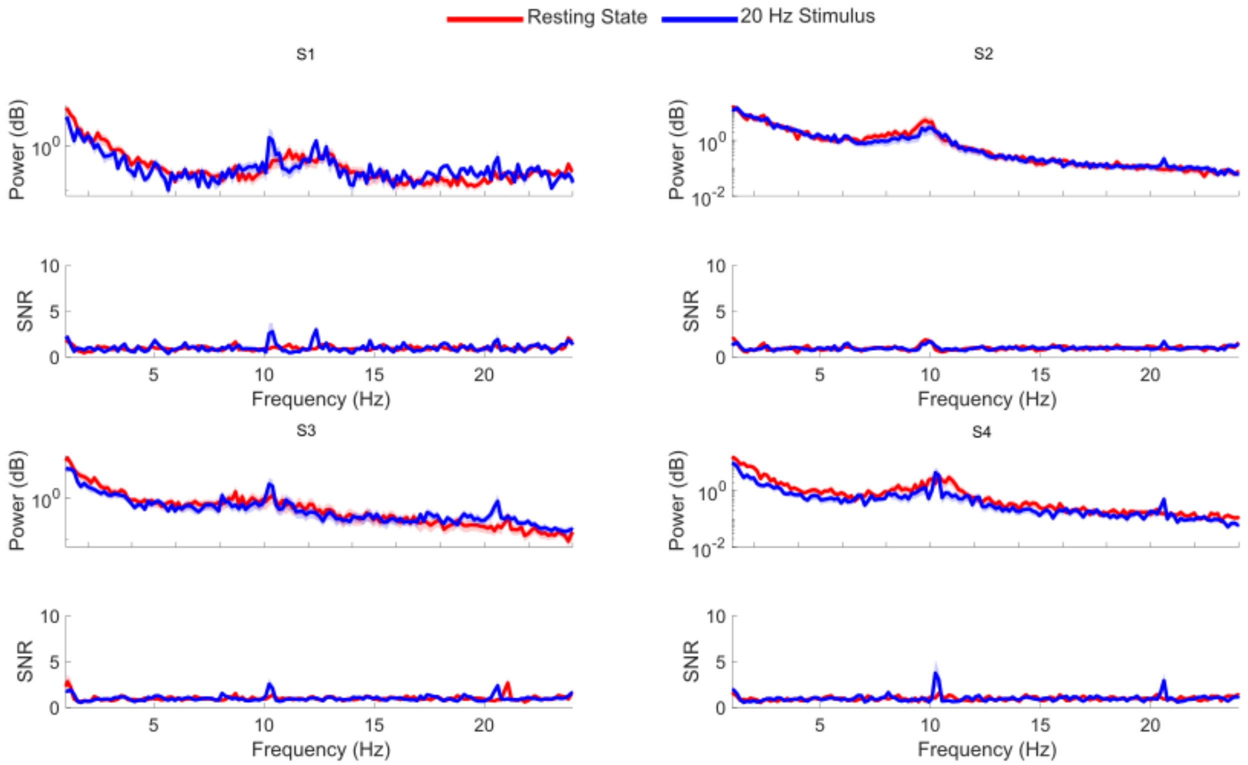


**Supplementary Figure S7**: Average SSVEP response during resting and stimulus recordings across occipital/parietal electrodes (P3, P4, O1, O2, Pz) for subjects S1 to S4. Resting state in red, SSVEP in blue.

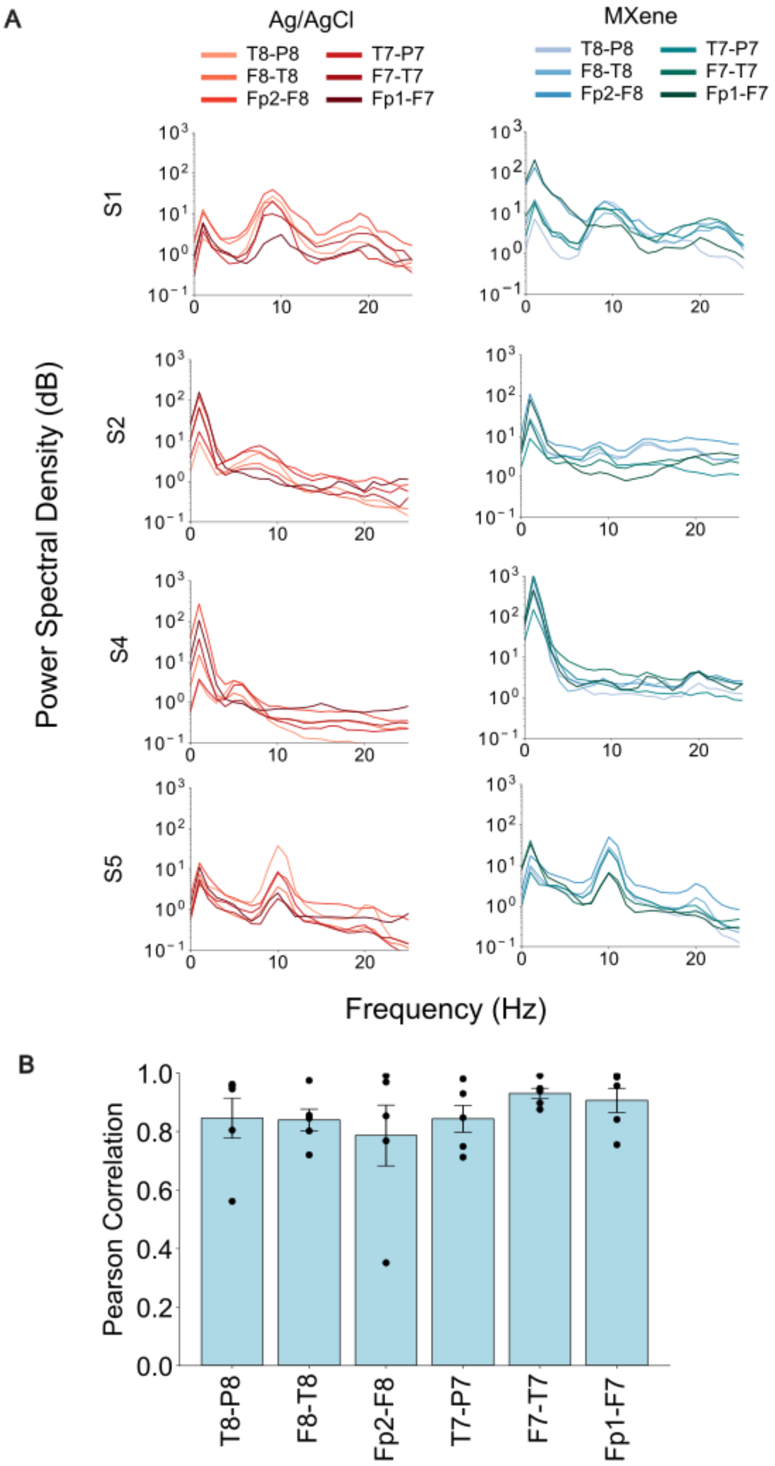


**Supplementary Figure S8.** (**A**) PSD for participants S1, S2, S4, and S5 during eyes closed task of the clinical EEG study. (**B**) Pearson correlation of the PSD of dry $Ti_3C_2T_x$ and gelled cup Ag/AgCl electrodes at different locations for all 5 participants.

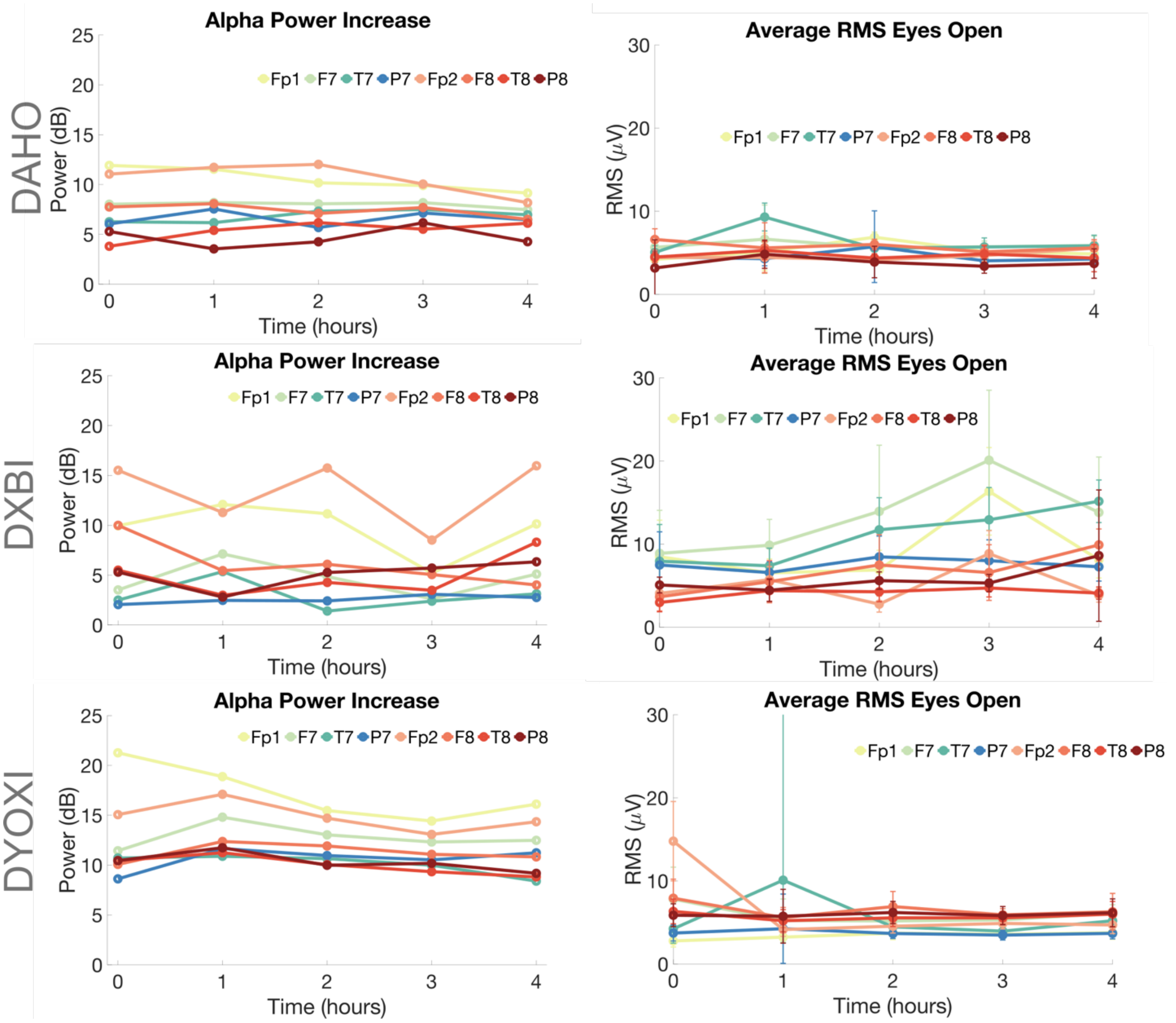


**Supplementary Figure S9.** Alpha power (8-13 Hz) difference between eyes close and eyes open and RMS during resting state eyes open for all other subjects over 4 hours.

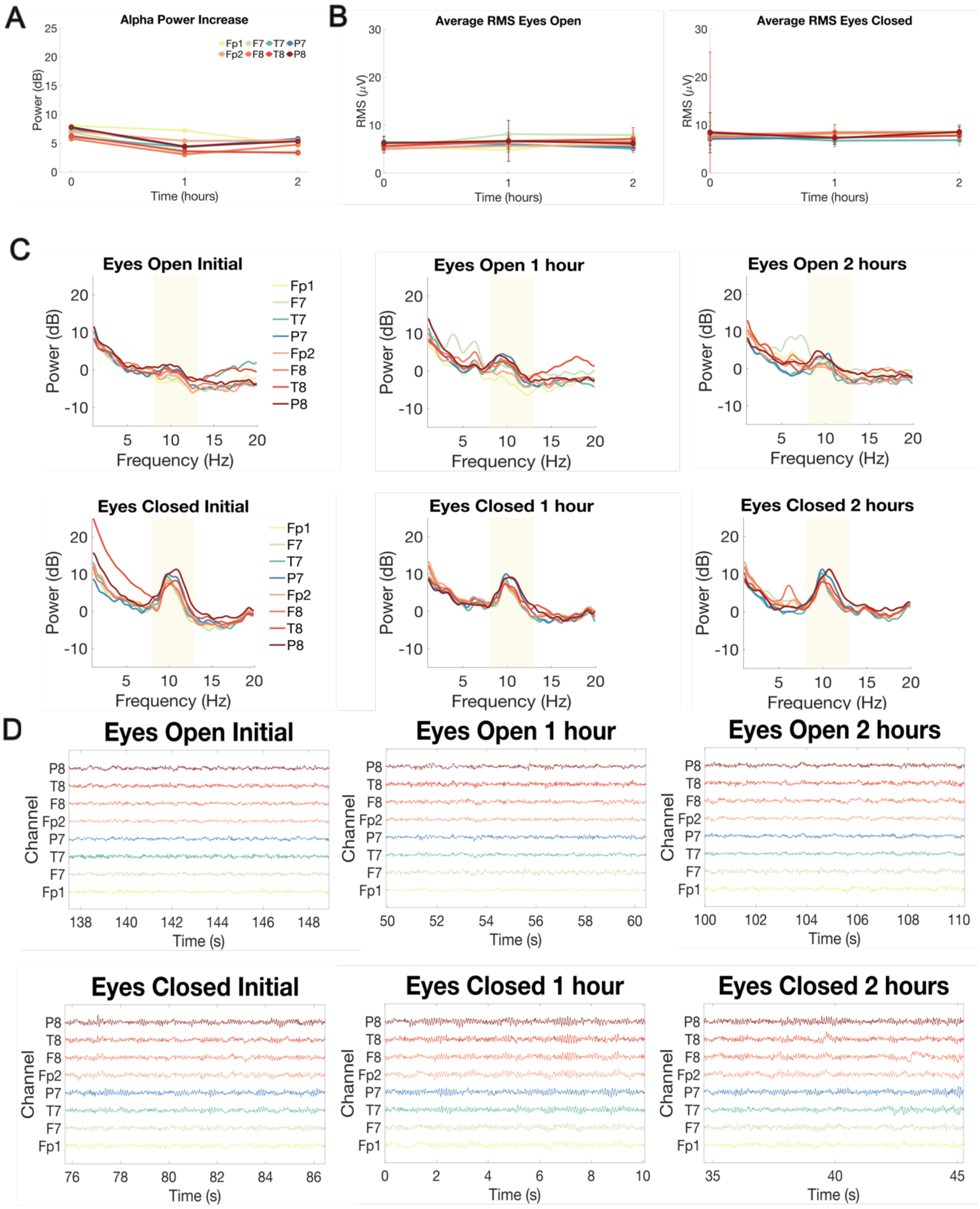


**Supplementary Figure S10.** Two-hour EEG recordings with no skin preparation for subject DYOX**. (A)** Difference in alpha-band power (8-13 Hz) between eyes-closed and eyes-open conditions for each electrode across 4 hours**. (B)** 2-s window averaged RMS amplitude of the signal on each electrode, separated by resting state condition. **(C)** Power spectral density estimate using 2-s, non-overlapping windows and (**D**) corresponding 10 s EEG traces for eyes-open (top) and eyes-closed (bottom) tasks for one subject.

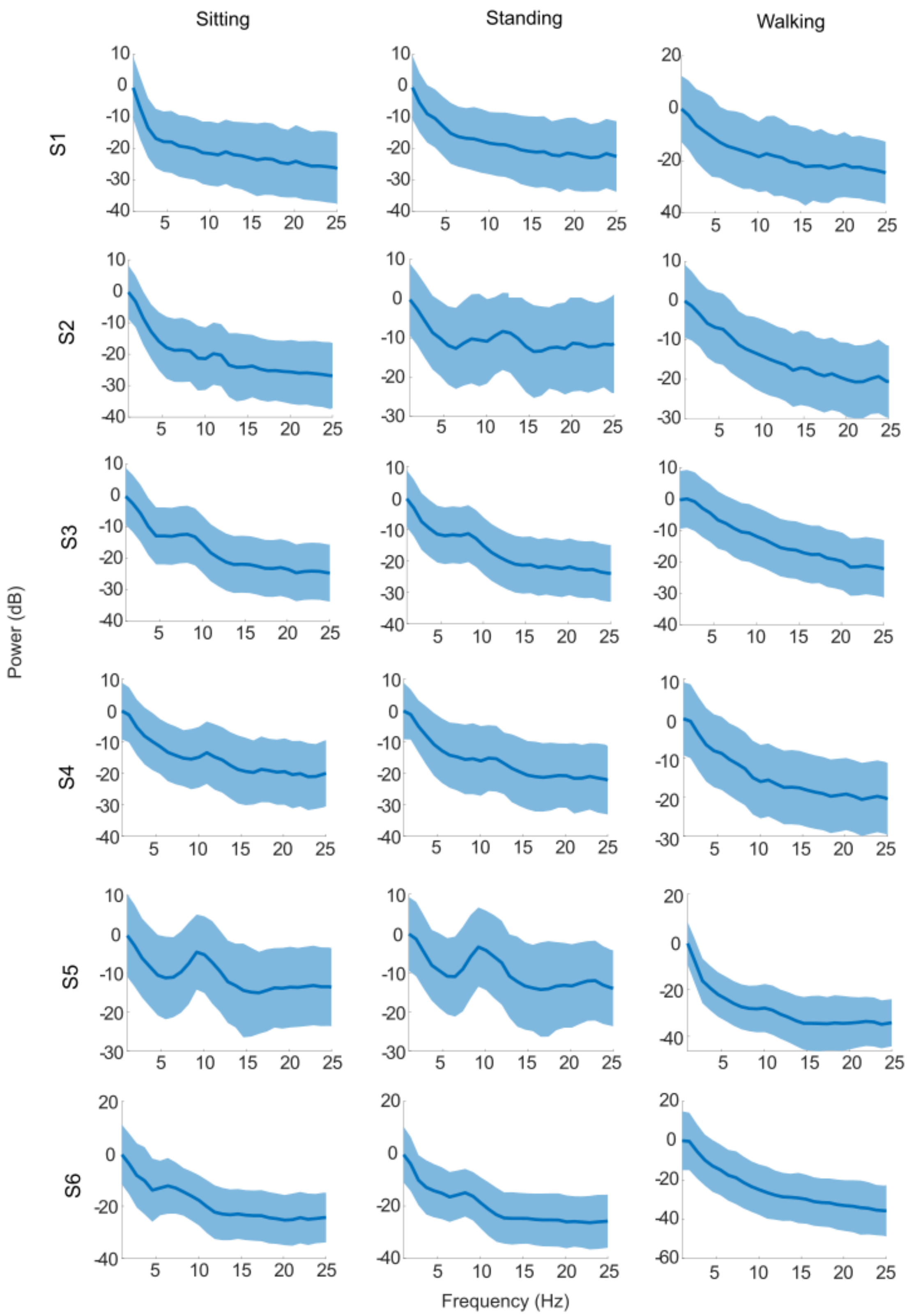


**Supplementary Figure S11**. Average power for the 8 dry $Ti_3C_2T_x$ electrodes during sitting, standing, and walking. Each row is a different participant.

**Supplementary Table S2**. Summary of the studies and EEG recording parameters.

| | Study 1 | Study 2 | Study 3 |
|---|---|---|---|
| **Electrodes** | 21 | 8 | 8 |
| **Recording Amplifier** | Bittium NeuroOne | Natus | Ripple Neuro Grapevine Trek |
| **Sampling Rate** | 5 kHz | 256 Hz | 30 kHz |
| **Location** | Drexel University | Hospital of the University of Pennsylvania | University of Pennsylvania |
| **Number of Participants** | 5 | 5 | 6 |

| **Race** | % of all participants |
|---|---|
| Asian | 21 |
| Caucasian | 58 |
| Native American | 5 |
| Black or African American | 16 |
| **Ethnicity** | |
| Hispanic/Latinx | 5 |
| Not Hispanic/Latinx | 95 |
| **Sex/Gender** | |
| Male | 42 |
| Female | 58 |

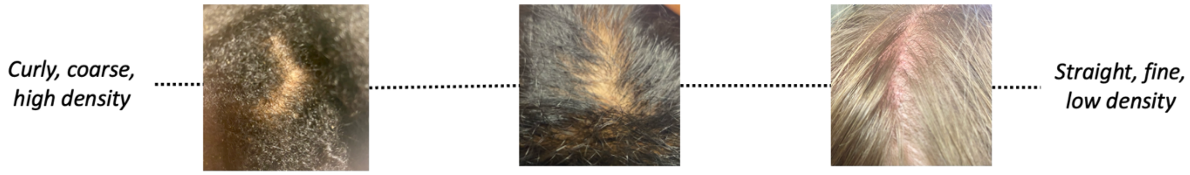


**Supplementary Figure S12**. Demographic information for participants across all studies.
